# Delineating the Switch between Senescence and Apoptosis in Cervical Cancer Cells under Ciclopirox Treatment

**DOI:** 10.3390/cancers13194995

**Published:** 2021-10-05

**Authors:** Anja L. Herrmann, Bianca J. Kuhn, Angela Holzer, Jeroen Krijgsveld, Karin Hoppe-Seyler, Felix Hoppe-Seyler

**Affiliations:** 1 Molecular Therapy of Virus-Associated Cancers, German Cancer Research Center (DKFZ), 69120 Heidelberg, Germany; anja.herrmann@dkfz.de (A.L.H.); a.holzer@dkfz.de (A.H.); 2 Faculty of Biosciences, Heidelberg University, 69120 Heidelberg, Germany; 3 Division of Proteomics of Stem Cells and Cancer, German Cancer Research Center (DKFZ), 69120 Heidelberg, Germany; bianca.kuhn@dkfz.de (B.J.K.); j.krijgsveld@dkfz.de (J.K.); 4 Medical Faculty, Heidelberg University, 69120 Heidelberg, Germany

**Keywords:** cervical cancer, human papillomavirus, senescence, apoptosis, therapy

## Abstract

**Simple Summary:**

Novel treatment options for cervical cancer are urgently required. Ciclopirox (CPX), an iron chelator, has shown promising anti-tumorigenic potential in several preclinical tumor models, including cervical cancer cells. In these cells, CPX can induce apoptosis, a form of cell death, or senescence, an irreversible cellular growth arrest. These different phenotypic outcomes may influence therapy response. Here, we show that the decision of cervical cancer cells to induce apoptosis or senescence is strongly dependent on glucose availability: CPX induces apoptosis under limited glucose availability, whereas under increased glucose supply, CPX treatment results in senescence. Further, we link the pro-apoptotic and pro-senescent activities of CPX to its capacity to block oxidative phosphorylation and to chelate iron, respectively. In addition, we show that the combined treatment of CPX and glycolysis inhibitors blocks the proliferation of cervical cancer cells in a synergistic manner. Collectively, we provide novel insights into the anti-proliferative activities of CPX in cervical cancer cells, elucidate the cellular decision between apoptosis or senescence induction, and provide a rationale to combine CPX with glycolysis inhibitors.

**Abstract:**

The iron-chelating drug ciclopirox (CPX) may possess therapeutic potential for cancer treatment, including cervical cancer. As is observed for other chemotherapeutic drugs, CPX can induce senescence or apoptosis in cervical cancer cells which could differently affect their therapy response. The present study aims to gain insights into the determinants which govern the switch between senescence and apoptosis in cervical cancer cells. We performed proteome analyses, proliferation studies by live-cell imaging and colony formation assays, senescence and apoptosis assays, and combination treatments of CPX with inhibitors of oxidative phosphorylation (OXPHOS) or glycolysis. We found that CPX downregulates OXPHOS factors and facilitates the induction of apoptosis under limited glucose availability, an effect which is shared by classical OXPHOS inhibitors. Under increased glucose availability, however, CPX-induced apoptosis is prevented and senescence is induced, an activity which is not exerted by classical OXPHOS inhibitors, but by other iron chelators. Moreover, we show that the combination of CPX with glycolysis inhibitors blocks cervical cancer proliferation in a synergistic manner. Collectively, our results reveal that the phenotypic response of cervical cancer cells towards CPX is strongly dependent on glucose availability, link the pro-apoptotic and pro-senescent activities of CPX to its bifunctionality as an OXPHOS inhibitor and iron chelator, respectively, and provide a rationale for combining CPX with glycolysis inhibitors.

## 1. Introduction

Approximately 5% of all cancer cases worldwide are attributed to infections with oncogenic types of human papillomaviruses (HPVs), such as HPV16 or HPV18 [1]. The most common HPV-induced malignancy is cervical cancer [2], which each year affects approximately 570,000 females and leads to over 300,000 cancer deaths [1]. Two viral oncoproteins, E6 and E7, play a crucial role in HPV-induced malignant cell transformation [3,4]. Sustained E6/E7 expression is required for maintaining the proliferation of HPV-positive tumor cells, and downregulation of E6/E7 typically leads to the rapid induction of senescence [5,6], a cellular state which is classically defined as an irreversible growth arrest [7]. Thus, agents that can block HPV E6/E7 expression are considered to possess potential for the therapeutic targeting of HPV-positive cancer cells [3].

We recently found that the iron chelator ciclopirox olamine (CPX) efficiently blocks HPV *E6/E7* oncogene expression, and acts anti-proliferative in cervical cancer cells [8]. CPX has been used clinically for decades as a topical antifungal agent for the treatment of mycoses of the skin, mucosa and nails, exhibiting an excellent pharmacological safety profile [9,10]. Notably, there has been an increasing interest in CPX to be possibly repurposed for cancer therapy [11], as it exerts anti-tumorigenic activities in a broad range of preclinical tumor models, including colorectal cancer [12,13], pancreatic cancer [14], breast cancer [15], cervical cancer [8], neuroblastoma [16], and hematologic malignancies [17]. Recently, clinical trials have been initiated employing a parenterally administrable CPX prodrug in bladder cancer patients [18].

Mechanistically, CPX targets multiple molecular pathways, which can mostly be attributed to the chelation of intracellular iron. Among others, CPX inhibits the enzymes ribonucleotide reductase [17] and deoxyhypusine hydroxylase [19], downregulates the cell cycle regulators cyclin D1 and E [15], and inhibits mTORC1 signaling [8,20]. Furthermore, potentially adding to their anti-tumorigenic effects, CPX and other iron chelators can inhibit mitochondrial oxidative phosphorylation (OXPHOS), probably due to decreased activity and expression of the iron-containing enzymes comprising the mitochondrial respiratory complexes [21,22].

At the phenotypic level, and in line with previous studies in other tumor models [11,23], we recently observed that CPX treatment of cervical cancer cells can result in apoptosis. Moreover, we found that CPX can also induce senescence, dependent on experimental conditions. Specifically, treatment of cervical cancer cells with CPX for 48–72 h programs the cells for the induction of senescence, whereas treatment for 72–96 h or longer determines the cells to undergo apoptosis [8]. This is reminiscent of the response of tumor cells towards established chemotherapeutic drugs, which also act via the induction of apoptosis and/or senescence [24], depending on experimental conditions, such as varying drug concentrations or the treated cell type [25,26]. These different phenotypic responses of cancer cells could be clinically relevant, as senescent cells have the potential to secrete factors that can exert pro-tumorigenic effects and increase therapy resistance (SASP: senescence associated secretory phenotype) [25,27]. Thus, it might be preferable to eliminate tumor cells by apoptosis rather than by inducing senescence. Yet, the knowledge of what determines whether a therapeutic agent preferably leads to senescence or apoptosis is surprisingly sparse, and mechanistic insights into these differential phenotypic responses are urgently required.

The present work aims to decipher determinants which govern the decision between the induction of senescence or apoptosis in HPV-positive cancer cells. We found that apoptosis induction by CPX is glucose-dependent and can be counteracted by increasing glucose availability, an effect which is also shared by other OXPHOS inhibitors. This indicates that reduced cellular energy supply is a key determinant for apoptosis induction after CPX treatment. On the other hand, we found that the potential of CPX to induce senescence is not impaired by increased glucose availability. Moreover, whereas the pro-senescent activity of CPX is not shared by other tested OXPHOS inhibitors, it is also observed for other iron chelators, indicating that the senescence-inducing activity of CPX is not linked to OXPHOS inhibition but to iron deprivation. Collectively, these findings provide new insights into the anti-tumorigenic mechanisms of CPX in cervical cancer cells and, more generally, also yield insights into the mechanisms underlying the decision between senescence and apoptosis induction in cancer cells.

## 2. Materials and Methods

### 2.1. Cell Culture and Treatment Conditions

HPV18-positive HeLa (RRID:CVCL_0030) and HPV16-positive SiHa (RRID:CVCL_0032) cervical cancer cells were obtained from the tumor bank of the German Cancer Research Center (DKFZ), Heidelberg. Cell lines were authenticated via SNP profiling (Multiplexion GmbH, Heidelberg, Germany), and tested negative for mycoplasma. HeLa mKate2 and SiHa mKate2 cells stably express the nuclear limited fluorescent mKate2 protein, and were generated from the cell lines mentioned above using the NucLight Red Lentivirus Reagent from Sartorius (Göttingen, Germany) according to the manufacturer’s protocol. All cells were cultivated at 37 °C, 21% O_2_, and 5% CO_2_ in DMEM (Gibco, Thermo Fisher Scientific, Waltham, MA, USA) containing 10% fetal bovine serum (Gibco, Thermo Fisher Scientific), 2 mM glutamine, 1 g/L glucose (if not specified otherwise), 100 U/mL penicillin, and 100 μg/mL streptomycin (Sigma-Aldrich, St. Louis, MO, USA). Stock cultures of mKate2 cells were additionally kept under selection with 1 µg/mL puromycin (Sigma-Aldrich).

The following drugs were used in the concentrations specified in the text:

Ciclopirox olamine (Santa Cruz Biotechnology, Dallas, TX, USA); antimycin A (Sigma-Aldrich); rotenone (MP Biomedicals, Santa Ana, CA, USA); metformin (Enzo Life Sciences, Lörrach, Germany); deferasirox (LKT Laboratories, St. Paul, MN, USA); deferoxamine mesylate (Sigma-Aldrich); dichloroacetate (Santa Cruz Biotechnology); 2-deoxy-D-glucose (Sigma-Aldrich); 6-aminonicotinamide (Cayman Chemical, Ann Arbor, MI, USA).

Ethanol (EtOH) was used as solvent control for CPX treatment with a maximum EtOH concentration of 0.1%, which did not affect cell growth or viability.

### 2.2. Mass-Spectrometric Proteome Analyses and GSEA Analyses

Mass spectrometry-based proteome analyses were performed in triplicates with SiHa cells treated with 10 µM CPX, or EtOH as solvent control for 48 h. A detailed description of sample preparation and quantitative proteome analyses by LC-MS/MS can be found elsewhere [28]. The proteomics data were deposited to the ProteomeXchange Consortium via the PRIDE [29] partner repository with the dataset identifier PXD011095. Gene set enrichment analysis (GSEA) was performed using GSEA v. 4.0.3 and gene sets included in the Molecular Signatures Database (MSigDB) version 7.0. Average log2FC values of all detected proteins were used as input for a pre-ranked enrichment analysis with the following parameters: number of permutations, 1000; enrichment statistic, weighted; max size, 500; min size, 15; normalization mode, meandiv.

### 2.3. Protein and RNA Analyses

Protein extraction and immunoblot analyses were performed as previously described [30]. The following primary antibodies were used:

Mouse anti-HPV16 E7 (NM2, gifted by Dr. Martin Müller, DKFZ, Heidelberg, Germany); chicken anti-HPV18 E7 (E7C) [31]; rabbit anti-Caspase 9 (cleaved Asp30) #7237 (Cell Signaling, Boston, MA, USA); mouse anti-COX4 (OXPHOS Complex IV subunit IV) #A21348 (Invitrogen, Thermo Fisher Scientific, Waltham, MA, USA); mouse anti-COX6B1 #393233 (Santa Cruz Biotechnology); mouse anti-Cyclin B1 #05-373 (Upstate, Sigma-Aldrich); mouse anti-Cyclin D1 #20044 (Santa Cruz Biotechnology); mouse anti-GPI #365066 (Santa Cruz Biotechnology); mouse anti-HKI #46695 (Santa Cruz Biotechnology); goat anti-HKII #6521 (Santa Cruz Biotechnology); mouse anti-NDUFS1 #271510 (Santa Cruz Biotechnology); mouse anti-NDUFS2 #390596 (Santa Cruz Biotechnology); rabbit anti-p21 #397 (Santa Cruz Biotechnology); mouse anti-p53 #126 (Santa Cruz Biotechnology); rabbit anti-phospho-p53 (Ser15) #9284 (Cell Signaling); mouse anti-p62 #610832 (BD Biosciences, Franklin Lakes, NJ, USA); mouse anti-cleaved-PARP (Asp214) #9546 (Cell Signaling); mouse anti-PFKP #514824 (Santa Cruz Biotechnology); rabbit anti-phospho-RPA32 (Ser33) #A300-246A (Bethyl Laboratories, Montgomery, TX, USA); mouse anti-β-Actin #47778 (Santa Cruz Biotechnology); mouse anti-Vinculin #73614 (Santa Cruz Biotechnology).

The following secondary antibodies were used: α-chicken IgG-HRP #2428 (Santa Cruz Biotechnology); α-goat IgG-HRP #2020 (Santa Cruz Biotechnology); α-mouse IgG-HRP #2005 (Santa Cruz Biotechnology); α-rabbit IgG-HRP #2004 (Santa Cruz Biotechnology).

Immunoblots were visualized using enhanced chemiluminescence (ECL Prime Western Blotting Detection Reagent, Cytiva, Marlborough, MA, USA and WesternBright Sirius HRP Substrate, Advansta, San Jose, CA, USA) and the Fusion SL Detection System (Vilber Lourmat, Eberhardzell, Germany). All immunoblot analyses were performed at least three times with consistent results. Uncropped original blots and quantifications of immunoblots can be found in Supplementary Figure S1.

RNA extraction and qRT-PCR analyses were performed as described in detail before [31]. In short, total cellular RNA was extracted using the column-based PureLink RNA Mini Kit (Invitrogen, Thermo Fisher Scientific) and reverse transcribed into cDNA with the ProtoScript^®^ II First Strand cDNA Synthesis Kit (NEB, Ipswich, MA, USA) according to the manufacturers’ instructions, using a mix of random and oligo dT primers. qRT-PCR analyses were performed with the SYBR Green PCR Master Mix (Applied Biosystems, Thermo Fisher Scientific) on a 7300 Real-Time PCR System Detector (Applied Biosystems, Thermo Fisher Scientific). Quantification was performed relative to 18S rRNA as internal reference, using the comparative Ct (2^−ΔΔCt^) method [32]. For statistical analysis, values were transformed logarithmically and fold change values were compared.

The following primer combinations were used for determining mRNA levels:

- 18S rRNA forward: 5’-CATGGCCGTTCTTAGTTGGT-3’ and
  18S rRNA reverse: 5’ ATGCCAGAGTCTCGTTCGTT-3’;
- Cyclin B1 forward: 5’-GCCTCTACCTTTGCACTTCCT-3’ and
  Cyclin B1 reverse 5’-TGTTGTAGAGTTGGTGTCCATT-3’;
- ID1 forward: 5’-AATCCGAAGTTGGAACCCCC-3’ and
  ID1 reverse: 5’-GAACGCATGCCGCCTCG-3’;
- IL1A forward: 5’-AACCAACGGGAAGGTTCTGA-3 and
  IL1A reverse: 5‘-AGGCTTGATGATTTCTTCCTCT-3’;
- IL6 forward: 5’-CCACCGGGAACGAAAGAGAA-3‘and
  IL6 reverse: 5’- CGAAGGCGCTTGTGGAGAA-3’;
- CDKN1A forward: 5’-GACCATGTGGACCTGTCACT-3’ and
  CDKN1A reverse: 5’-GCGGATTAGGGCTTCCTCTT-3’;
- SERPINE1 forward: 5’-GACCGCAACGTGGTTTTCTC-3’ and
  SERPINE1 reverse: 5’-GCCATGCCCTTGTCATCAAT-3’.

### 2.4. Live-Cell Imaging

All live-cell imaging experiments were performed using the IncuCyte^®^ S3 device (Sartorius) with HeLa mKate2 and SiHa mKate2 cells. Three thousand cells were seeded per well in a 96-well plate and 48 h later cells were treated as detailed in the text. Every 4 h, four images per well were acquired at a magnification of 10×. For proliferation analyses, over 6 days viable cell numbers were determined by counting labelled nuclei using the IncuCyte^®^ 2019B software. For detection of cell death, the IncuCyte^®^ Cytotox Green Dye (Sartorius, Cat. No. 4633) was used at a concentration of 100 nM. Over 6 days, the number of dead cells per well was determined by counting green fluorescent objects and was normalized to cell confluence to account for different proliferation rates.

### 2.5. Apoptosis Assays

For detecting apoptosis, TUNEL (terminal deoxynucleotidyltransferase-mediated UTP end labeling) assays were performed using the In Situ Cell Death Detection Kit (Roche, Basel, Switzerland), with cells grown on coverslips according to the manufacturer’s instructions. At least five images per coverslip were analyzed with a Cell Observer microscope (Zeiss, Jena, Germany), and the percentage of TUNEL positive cells was determined using an ImageJ macro (Damir Krunic, Light Microscopy Core Facility, DKFZ, Heidelberg, Germany) in relation to the total cell count determined by DAPI (Cell Signaling Technology) staining.

Cleaved PARP and cleaved caspase 9 levels were analyzed by immunoblotting.

### 2.6. Colony Formation and Senescence Assays

For colony formation assays, cells were treated for the indicated time, then split, re-plated in a drug-free medium, and cultivated for the time periods described in the text. Colonies were then fixed and stained with formaldehyde-crystal violet. Colony area and, where applicable, colony count were quantified using an ImageJ macro (Damir Krunic, Light Microscopy Core Facility, DKFZ) (Supplementary Figure S2).

Senescence assays were performed in parallel to colony formation assays. Cells were treated, split, and re-plated, and after 4 days of release, cells were stained for SA-β-gal activity as described by Dimri et al. [33]. Images were acquired using the EVOSxl Core Cell Imaging System (Invitrogen, Thermo Fisher Scientific) with 20× magnification.

### 2.7. Combination Index Analyses

Combination index analyses were performed using the CompuSyn software (ComboSyn, Inc., Paramus, NJ, USA) according to the Chou–Talalay method, which is based on the median-effect equation [34]. Proliferation curves of cells treated with the single drugs or the drug combination (in a constant ratio) were determined as described in 2.4. Combination indices were calculated using the area under the curve values of the proliferation curves up to 5 days of treatment. CI < 1, CI = 1, and CI < 1 indicate antagonistic, additive, and synergistic effects, respectively.

### 2.8. Statistical Analyses

SigmaPlot version 14.0 (Systat Software Inc., San Jose, CA, USA) was used for statistical tests. For the comparison of relative mRNA levels after CPX treatment, a one-sample *t*-test was performed with a test mean of zero. Shapiro–Wilk normality analysis was performed with an alpha-value of 0.05. For comparison of TUNEL-positive cell percentages, a two-sided Student’s *t*-test was used. Statistical significance was assumed for *p*-values ≤ 0.05 (*), ≤ 0.01 (**), ≤ 0.001 (***).

## 3. Results

### 3.1. CPX Regulates Factors Involved in Oxidative Phosphorylation and Glycolysis

Enzymatic complexes comprising the mitochondrial electron transport chain (ETC) are known to be iron-dependent, as they require iron atoms for proper conformation, and iron in the form of hemes and iron-sulfur clusters as electron acceptors and donors [35]. Moreover, iron chelation can decrease the expression of several proteins comprising OXPHOS complexes [36].

**Figure 1:**
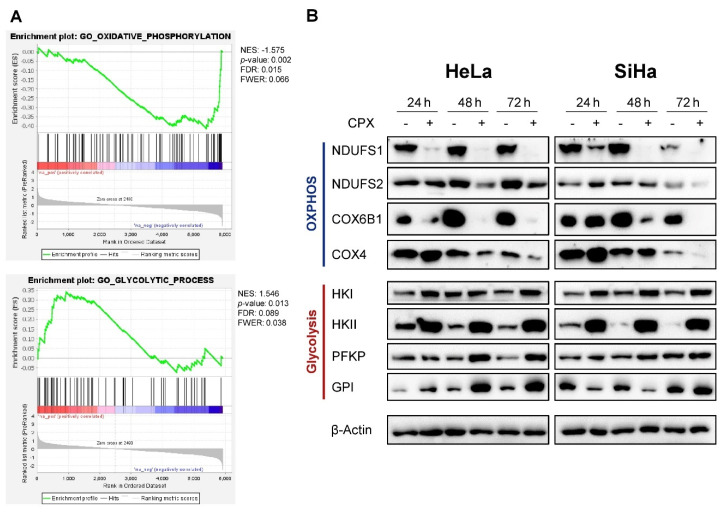
Ciclopirox (CPX) downregulates oxidative phosphorylation (OXPHOS)-related factors and upregulates glycolysis-related factors in cervical cancer cells. (**A**) Proteome analyses of SiHa cells treated with 10 µM CPX for 48 h. Experiments were performed in triplicate, and data were analyzed via gene set enrichment analysis (GSEA). Enrichment plots of the gene sets “GO_glycolytic_process” and “GO_oxidative_phosphorylation” are shown. NES: normalized enrichment score; FDR: false discovery rate; FWER: family-wise error rate. (**B**) Immunoblot analyses of HeLa or SiHa cells treated for 24, 48, or 72 h with 10 µM CPX (+) or solvent control EtOH (-), assessing protein levels of exemplary factors involved in OXPHOS or glycolysis. NDUFS1, NDUFS2: NADH:Ubiquinone oxidoreductase core subunits S1 and S2; COX6B1, COX4: cytochrome c oxidase subunits 6B1 and 4. HKI and HKII: hexokinase I and II; PFKP: phosphofructokinase; GPI: glucose-6-phosphate isomerase; β-Actin: representative loading control.

We performed mass spectrometry-based quantitative proteome analyses to assess the relative changes in protein abundances in HPV16-positive SiHa cervical cancer cells under CPX treatment. Gene set enrichment analyses (GSEA) revealed that CPX negatively enriches factors involved in OXPHOS (Figure 1A, left panel and Supplementary Figure S3A). Concomitantly, an upregulation of glycolysis-related factors was observed (Figure 1A, right panel and Supplementary Figure S3B), indicating that the CPX-treated cells attempt to compensate the reduced energy production via OXPHOS with an increase in glycolysis, in line with the metabolic plasticity of cancer cells [37].

This notion is further supported by immunoblot analyses of HPV18-positive HeLa and HPV16-positive SiHa cervical cancer cells, which show that CPX treatment accordingly affects the protein levels of exemplary factors involved in OXPHOS or glycolysis (Figure 1B). Expression levels of the mitochondrial respiratory chain subunits NADH:Ubiquinone oxidoreductase core subunits S1 and S2 (NDUFS1, NDUFS2; complex I) and cytochrome c oxidase subunits 6B1 and 4 (COX6B1, COX4; complex IV) are decreased after up to 72 h treatment with CPX, whereas expression levels of enzymes of the glycolytic pathway, such as hexokinase I and II (HKI, HKII), phosphofructokinase (PFKP), and glucose-6-phosphate isomerase (GPI), are increased. In SiHa cells, the upregulation of PFKP is not as pronounced as in HeLa cells, and GPI is initially downregulated and upregulated only after 72 h. This indicates, to some degree, a cell type dependent regulation for these factors, possibly due to metabolic differences between the two cell lines.

### 3.2. Increased Glucose Availability Protects Cells against CPX-Induced Apoptosis

These findings raise the possibility that the pro-apoptotic effects induced by CPX in HPV-positive cells [8] could (at least partially) be due to OXPHOS inhibition and subsequent energy scarcity. This would be consistent with our observation that longer treatment of cervical cancer cells, which is accompanied by an increasing limitation of glucose availability, promotes apoptosis induction by CPX [8]. We therefore investigated the phenotypic effects of CPX treatment on HeLa or SiHa cells cultivated under varying glucose concentrations.

**Figure 2:**
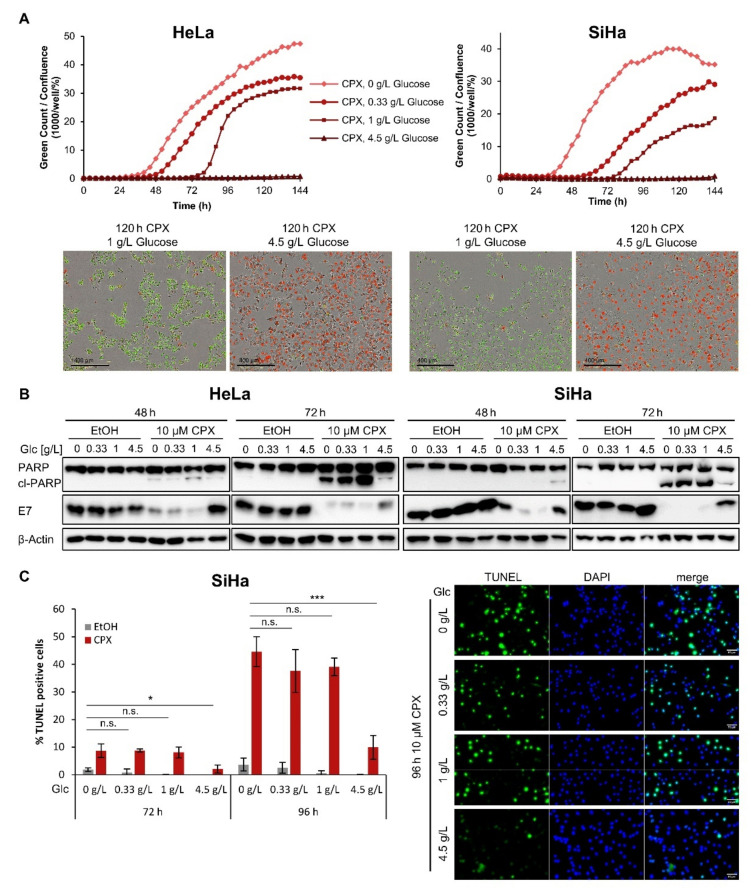
Increased glucose availability protects cells against CPX-induced apoptosis. (**A**) For the quantification of cell death, HeLa mKate2 or SiHa mKate2 cells were treated for up to 144 h with 10 µM CPX under the indicated glucose levels in the presence of 100 nM IncuCyte^®^ Cytotox Green Reagent. Images were acquired every 4 h and dead cells were quantified as green counts per well and normalized to the confluence in percent (upper panels). Exemplary images after 120 h of treatment are shown, viable cells can be identified by red labelled nuclei, dead cells fluoresce green due to Cytotox activation (lower panels). Scale bars: 400 µm. Glc: glucose. (**B**) Immunoblot analyses of PARP, cleaved PARP (cl-PARP), and HPV18 or HPV16 E7 expression levels in HeLa or SiHa cells, respectively, treated with 10 µM CPX or solvent control (EtOH) for 48 or 72 h in the presence of the indicated amounts of glucose. β-Actin: representative loading control. (**C**) Quantification of the percentage of TUNEL positive SiHa cells after 72 or 96 h treatment with solvent control or 10 µM CPX under the indicated glucose levels (left panel). The average of three replicates is shown, error bars represent standard deviations. n.s.: non-significant; * = *p* ≤ 0.05; *** = *p* ≤ 0.001. Representative images of the TUNEL assays (right panel) depict cells after 96 h CPX treatment. Scale bars: 50 µm.

In line with our previous results [8], cervical cancer cells cultivated at 1 g/L glucose die after 72–96 h of CPX treatment, as indicated by increased cytotoxicity assessed in live-cell imaging analyses. Treating cells with CPX under lower glucose availability (0.33 g/L) or in the absence of glucose leads to an earlier and more pronounced onset of cell death (Figure 2A). In contrast, increased levels of glucose (4.5 g/L) efficiently protect cells from CPX-induced cell death. Collectively, these findings indicate a strong glucose dependency of the phenotypic response of cervical cancer cells towards CPX, in that limited glucose availability facilitates and higher glucose availability counteracts CPX-induced cell death, respectively.

To verify that this glucose-dependent form of CPX-induced cell death is apoptosis, we performed immunoblot analyses of the apoptosis marker cl-(cleaved-)PARP (poly(ADP-ribose)polymerase). We observed a strong accumulation of cl-PARP in cervical cancer cells after 72 h of CPX treatment under lower glucose concentrations (0 to 1 g/L), but not under higher glucose concentrations (4.5 g/L) (Figure 2B). Moreover, the CPX-induced downregulation of the HPV oncoprotein E7 is counteracted by an increased glucose supply, which has similarly been observed for hypoxia- or metformin-induced repression of the HPV oncogenes [28,30].

As an additional method for apoptosis detection, we performed TUNEL (terminal deoxynucleotidyltransferase-mediated dUTP-biotin nick end labeling) analyses [38] of CPX-treated SiHa cells. In line with the results of the cytotoxicity assays and the changes of cl-PARP expression, the substantial decrease in TUNEL-positive cells also demonstrates the protective effect of increased glucose availability against CPX-induced apoptosis (Figure 2C).

### 3.3. Increased Glucose Availability Favors Induction of a Senescent Phenotype under CPX Treatment

**Figure 3:**
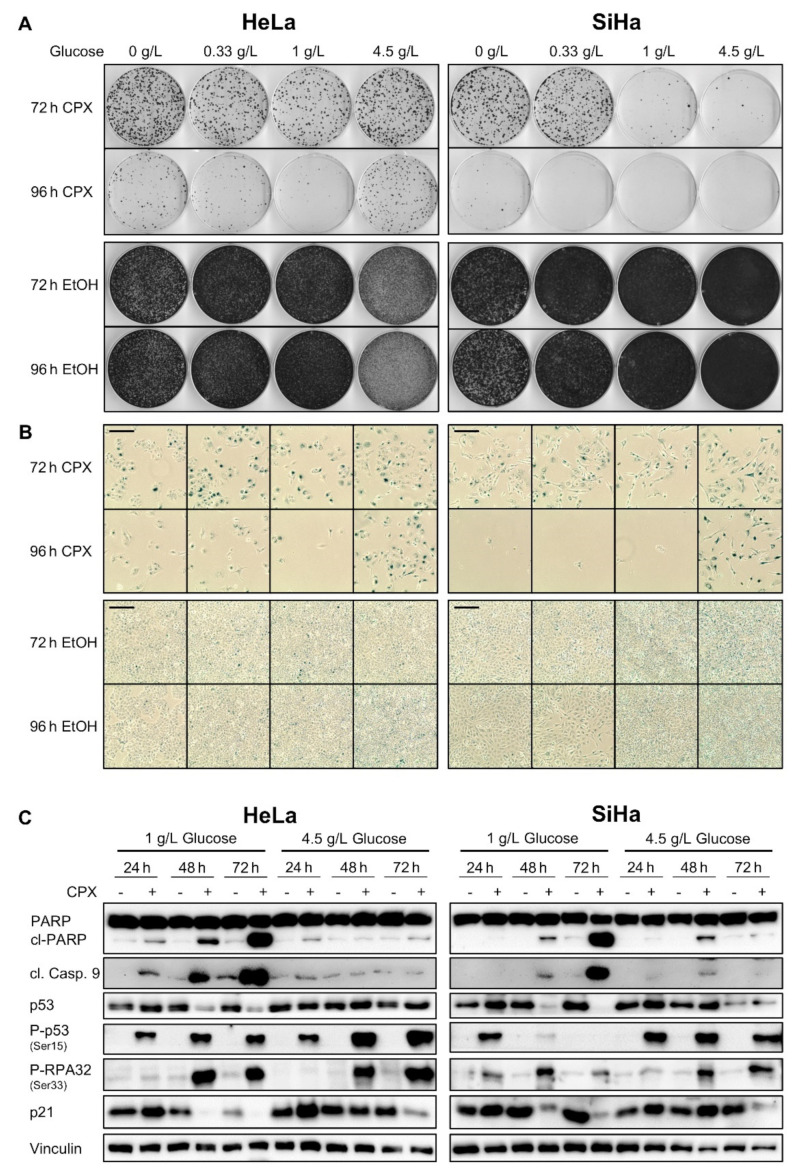
Increased glucose availability favors development of a senescent phenotype under long-term CPX treatment. (**A**) Colony formation assays (CFAs) of HeLa and SiHa cells treated with 10 µM CPX or EtOH as solvent control for 72 or 96 h under the indicated glucose concentrations. Subsequently, cells were grown in CPX-free medium under 1 g/L glucose for 12 days, fixed and stained. (**B**) Senescence assays of HeLa and SiHa cells treated with 10 µM CPX or EtOH as solvent control for 72 or 96 h under the indicated glucose concentrations. Subsequently, cells were cultivated for 4 days in CPX-free medium before SA-β-gal assays were performed. Scale bars: 200 µm. (**C**) Immunoblot analysis of HeLa and SiHa cells treated for 24, 48, or 72 h with 10 µM CPX (+) or solvent control (-), analyzing protein levels of PARP and cl-PARP, cleaved caspase 9 (cl. Casp. 9), total p53, p53 phosphorylated at serine 15, RPA32 phosphorylated at serine 33, and p21 upon cultivation under 1 g/L or 4.5 g/L glucose. Vinculin: representative loading control.

As shown above, CPX-treated cervical cancer cells are protected from apoptosis under increased glucose availability (4.5 g/L). Yet, we found that these cells are still inhibited in their proliferation capacity in colony formation assays (CFAs) (Figure 3A, upper panels) when compared with untreated cells (Figure 3A, lower panels).

Notably, long-term CPX treatment under higher glucose availability (72–96 h, 4.5 g/L) leads to typical morphological characteristics of senescence in HPV-positive cancer cells, such as enlargement, flattening, and cytoplasmic extensions [31] (Figure 3B). Since senescence is an irreversible growth arrest, this explains the observed reduction of colony formation capacity in the absence of apoptotic markers. The induction of senescence under these experimental conditions is further corroborated by positive staining of the cells for the well-established senescence marker senescence-associated β-galactosidase (SA-β-gal) (Figure 3B). Cells cultivated under lower glucose concentrations (1.0 g/L, 0.33 g/L) or in the absence of glucose also undergo senescence after CPX treatment for 72 h, however, under prolonged treatment (96 h) they increasingly die, as indicated by the reduced number of surviving cells visible in the senescence assays. CPX treatment for shorter periods (24 or 48 h) leads to less efficient senescence induction and to increased clonal survival when compared with more prolonged treatment (72 or 96 h) (Supplementary Figure S4).

These results suggest that glucose availability is a decisive factor in determining whether CPX-treated cells enter a senescent or apoptotic state. To investigate this phenomenon in further detail, we compared the expression of factors linked to senescence and/or apoptosis in cervical cancer cells cultivated under 1 g/L or 4.5 g/L glucose (Figure 3C). In line with the results described above, apoptosis markers such as cleaved caspase 9 and cl-PARP, are induced by CPX under 1 g/L, but not under 4.5 g/L glucose. Total p53 levels are reduced by CPX under 1 g/L, but not under 4.5 g/L glucose. In contrast, phosphorylated p53 (Ser15) and RPA32 (Ser33), two markers indicating DNA damage [39,40], are upregulated under both 1 g/L and 4.5 g/L glucose, in line with findings that CPX possesses genotoxic potential [41]. Interestingly, p21, which is regarded as a key factor for senescence induction [7], is repressed after 48 h treatment under 1 g/L glucose, while increased glucose levels (4.5 g/L) counteract this downregulation (Figure 3C). This may hint at a role for p21 in determining the switch towards senescence under CPX treatment. In contrast to p21, downregulation of p62 (SQSTM1), which is regarded as crucial step for SASP development [42], occurs under both tested glucose conditions, similarly to inhibition of the cell cycle regulator Cyclin D1, whereas effects on Cyclin B1 expression are less pronounced (Supplementary Figure S5). Investigations at the mRNA level indicate that the expression of selected senescence-associated genes is shifted by CPX treatment into a pro-senescent direction under both examined glucose concentrations (Supplementary Figure S6). Expression of genes coding for key SASP components such as *IL1A* and *IL6* [43] is upregulated, as is the expression of the pro-senescent [44,45] *CDKN1A* (p21) and *SERPINE1* (PAI-1) genes. In contrast, the anti-senescent [46,47] genes *ID1* and *CCNB1* (Cyclin B1) are downregulated upon CPX treatment.

### 3.4. The Glucose-Dependent Apoptosis Induction through CPX Is Shared by Other OXPHOS Inhibitors

To explore if this glucose dependency of apoptosis induction is linked to the ability of CPX to interfere with OXPHOS, we compared the effects of CPX with those of well-characterized inhibitors of OXPHOS, namely rotenone (complex I inhibitor), antimycin A (complex III inhibitor), and metformin (complex I inhibitor).

**Figure 4:**
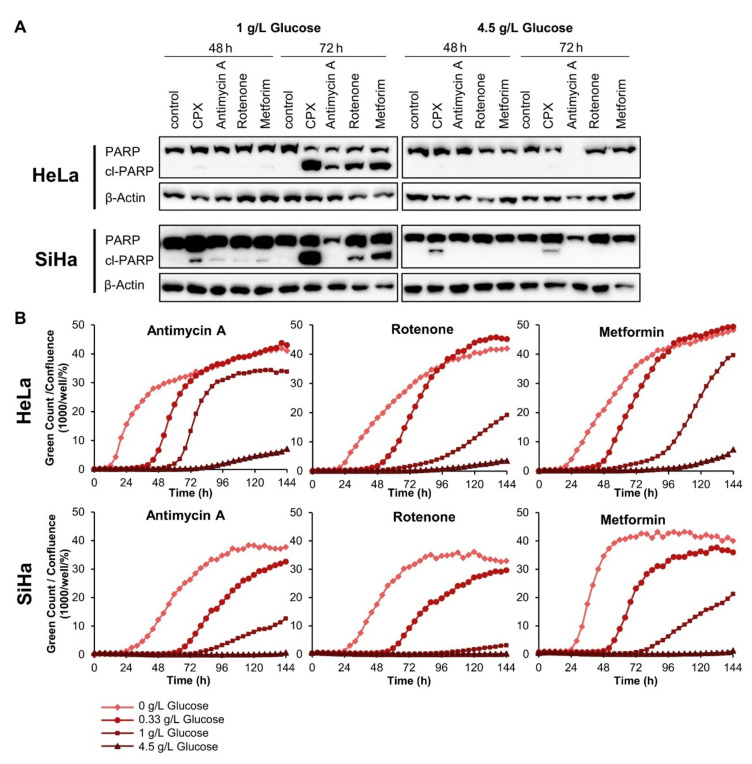
OXPHOS inhibitors induce glucose-dependent apoptosis in cervical cancer cells. (**A**) Immunoblot analyses of PARP and cl-PARP levels in HeLa or SiHa cells after 48 or 72 h of treatment with the indicated OXPHOS inhibitors under 1 g/L or 4.5 g/L glucose. The following drug concentrations were used: 10 µM CPX; 5 nM (HeLa)/ 10 nM (SiHa) antimycin A; 20 nM rotenone; 2.5 mM (HeLa)/ 7.5 mM (SiHa) metformin. β-Actin: loading control. (**B**) HeLa mKate2 (upper panels) or SiHa mKate2 (lower panels) cells were treated for 144 h with OXPHOS inhibitors under the indicated glucose concentrations in the presence of 100 nM IncuCyte^®^ Cytotox Green Reagent. Green counts indicating dead cells were quantified every 4 h by live-cell imaging, and normalized to cell confluence. The following drug concentrations were used: 10 nM antimycin A; 20 nM rotenone; 2.5 mM (HeLa)/ 5 mM (SiHa) metformin.

We found that the three latter compounds also induce apoptosis in cervical cancer cells, as indicated by increased PARP cleavage and enhanced cytotoxicity in live-cell imaging analyses. Both rotenone and metformin treatment lead to a robust increase in PARP cleavage after 72 h under 1 g/L glucose (Figure 4A, left panels). As observed for CPX (Figure 2B), this effect is again strongly counteracted for both drugs by supplying the cells with 4.5 g/L glucose (Figure 4A, right panels). Antimycin A also leads to increased cl-PARP amounts in HeLa cells at 1 g/L glucose. This, however, is not detectable in SiHa cells, possibly due to the fact that antimycin A can lead to a substantial downregulation of total PARP amounts (Figure 4A). Similar to CPX (Figure 2A), all three OXPHOS inhibitors induce cell death under 1 g/L glucose, as visualized by cytotoxicity assays (Figure 4B). This response of cervical cancer cells towards the OXPHOS inhibitors is further facilitated under decreased glucose availability (0.33 g/L) or in the absence of glucose. In contrast, increased glucose availability (4.5 g/L) efficiently blocks the induction of cell death.

### 3.5. The Pro-Senescent Activity of CPX Is Shared by Other Iron Chelators, but Not by Other OXPHOS Inhibitors

**Figure 5:**
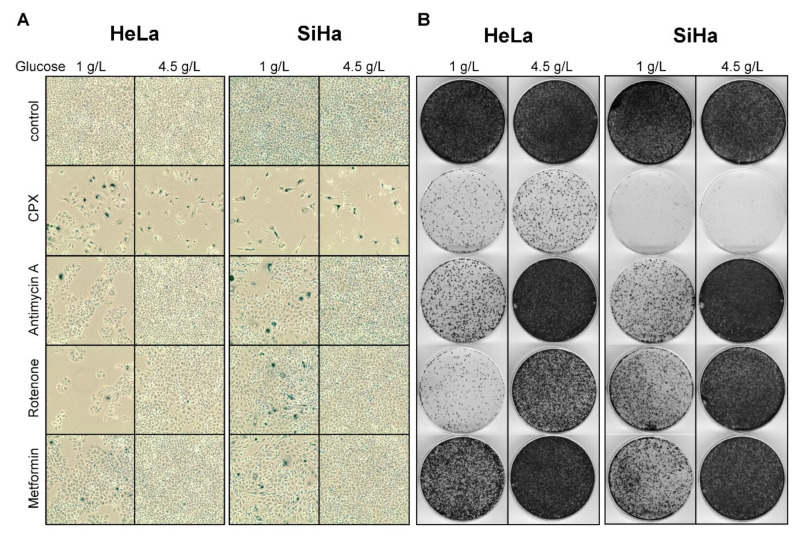
The capacity of CPX to induce senescence under increased glucose availability is not shared by other OXPHOS inhibitors. (**A**) Senescence assays of HeLa or SiHa cells treated with the indicated OXPHOS inhibitors for 72 h under 1 g/L or 4.5 g/L glucose. Before SA-β-gal assays were performed, cells were released in drug-free medium (1 g/L glucose) for 4 days. The following drug concentrations were used: 10 µM CPX; 2 nM (HeLa)/ 5 nM (SiHa) antimycin A; 20 nM rotenone; 2.5 mM (HeLa)/ 7.5 mM (SiHa) metformin. Scale bar: 200 µm. (**B**) Concomitant CFAs to (**A**), cells were released in drug-free medium (1 g/L glucose) for 11 days before fixing and staining colonies.

Interestingly, whereas under CPX-treatment many cells exhibit typical morphological signs of senescence and stain positive for SA-β-gal, after treatment with rotenone, antimycin A, or metformin only few cells stain SA-β-gal positive under 1 g/L glucose and no SA-β-gal positive cells are observed under 4.5 g/L glucose (Figure 5A). Thus, in contrast to CPX, all other tested OXPHOS inhibitors only weakly induce senescence in cervical cancer cells under limited glucose supply and completely lack pro-senescent activity under higher glucose availability.

The lack of senescence induction in cervical cancer cells by the OXPHOS inhibitors antimycin A, rotenone, or metformin in the presence of increased glucose availability is further corroborated by the CFAs performed in parallel. As senescence is an irreversible growth arrest, cells which become senescent should no longer be able to form colonies upon release from treatment. After treatment for 72 h under 4.5 g/L glucose and subsequent drug release, we found that only CPX treatment, but not treatment with either antimycin A, rotenone, or metformin, strongly blocks the colony formation potential of cervical cancer cells (Figure 5B).

**Figure 6:**
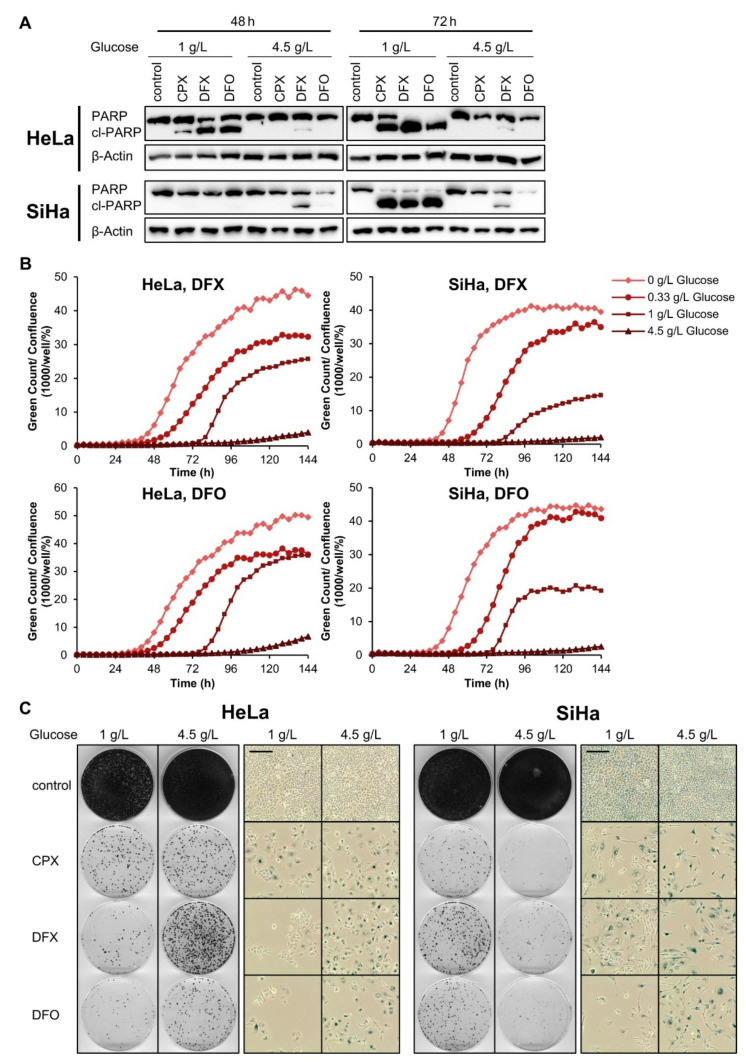
Iron chelators induce apoptosis in a glucose-dependent manner and act pro-senescent under increased glucose availability. (**A**) Immunoblot analyses of PARP and cl-PARP levels in HeLa or SiHa cells, treated with the indicated iron chelators under 1 g/L or 4.5 g/L glucose for 48 or 72 h. β-Actin: representative loading control. (**B**) Cytotoxicity assays of HeLa mKate2 or SiHa mKate2 cells treated with deferasirox (DFX) or deferoxamine (DFO) over the course of 144 h under the indicated glucose concentrations. Green object count indicating the number of dead cells was assessed every 4 h and normalized to cell confluence. (**C**) CFAs and concomitant senescence assays of HeLa or SiHa cells treated with the indicated iron chelators for 72 h under 1 g/L or 4.5 g/L glucose. After treatment, cells were split and cultured in drug-free medium (containing 1 g/L glucose) for further 4 days (senescence assays) or 13 days (CFAs). Scale bars: 200 µm. In Figure 6A–C, the following drug concentrations were used: 10 µM CPX; 50 µM DFX; 100 µM DFO.

These findings raise the question whether the difference in senescence regulation between CPX and the other tested OXPHOS inhibitors is linked to the iron-chelating properties which are unique to CPX. We therefore comparatively analyzed CPX and two iron chelators which are structurally unrelated to CPX, deferasirox (DFX) and deferoxamine (DFO). As seen for CPX as well as for rotenone, antimycin A, and metformin (Figure 4 and Figure 5), DFO and DFX induce apoptosis in a glucose-dependent manner (Figure 6A–C). This effect is again efficiently counteracted by increasing glucose availability, as indicated by decreased PARP cleavage (Figure 6A) and decreased cytotoxicity at 4.5 g/L glucose (Figure 6B). Notably, however, similar to CPX—and in strong contrast to rotenone, antimycin A, or metformin (Figure 5)—both DFO and DFX also induce senescence in cervical cancer cells under 4.5 g/L glucose, as indicated by positive staining for SA-β-gal activity and inhibition of cellular proliferation in colony formation assays upon drug release (Figure 6C). Collectively, these results indicate that senescence induction by CPX, in contrast to apoptosis induction, is not due to its capacity to inhibit OXPHOS, but is linked to its iron-chelating activity.

### 3.6. CPX Synergizes with Glycolysis Inhibitors

In view of our data indicating that the pro-apoptotic effect of CPX is related to its activity as an OXPHOS inhibitor, we hypothesized that CPX may synergize with glycolysis inhibitors, which could enable a significant dose reduction of both drugs.

**Figure 7:**
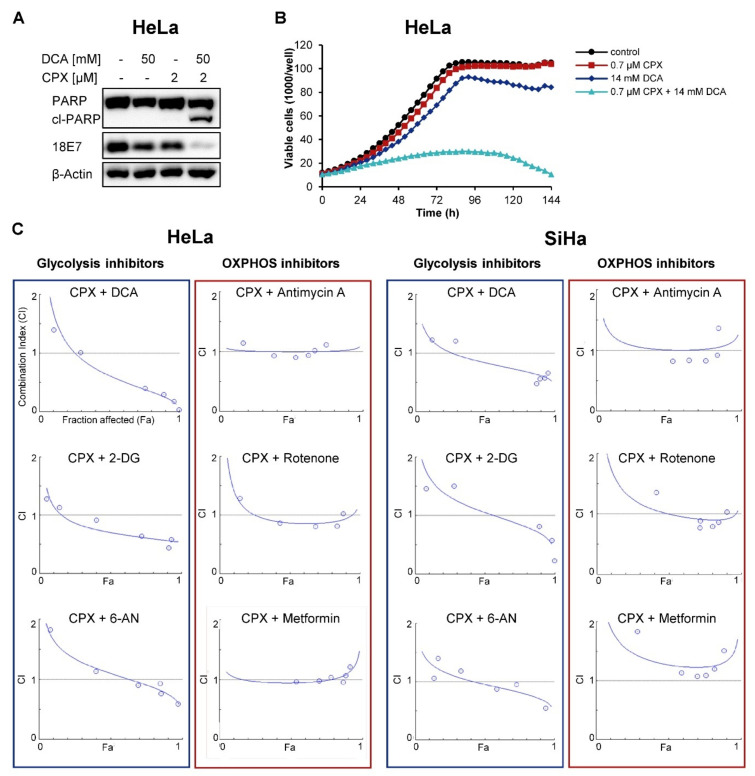
CPX acts synergistically with glycolysis inhibitors. (**A**) Immunoblot analyses of PARP, cl-PARP, and HPV18 E7 levels in HeLa cells after 48 h treatment with CPX and DCA as indicated. β-Actin: loading control. (**B**) Growth curves of HeLa mKate2 cells treated with the indicated doses of CPX and DCA. Red object count indicating viable cells was assessed every 4 h over a period of 144 h using the IncuCyte^®^ S3 live-cell imaging system. (**C**) HeLa mKate2 or SiHa mKate2 cells were treated with varying doses of CPX and the indicated OXPHOS or glycolysis inhibitors with a constant drug ratio over the course of 5 days. Every 4 h, viable cell count was assessed. Fraction affected (Fa) vs. combination index (CI) plots were created with the CompuSyn software, based on viable cell count. Data from one representative experiment each are illustrated. All experiments depicted in Figure 7 were performed under a concentration of 1 g/L glucose.

The drug dichloroacetate (DCA) inhibits the enzyme pyruvate dehydrogenase kinase 1 (PDK1) and induces a shift of pyruvate metabolism from glycolysis towards OXPHOS, resulting in a reversal of the Warburg effect [48]. Notably, whereas low doses of either CPX or DCA do not have appreciable effects on PARP cleavage or E7 protein levels, the same doses applied in combination induce PARP cleavage, indicating apoptosis induction, and lead to E7 downregulation in HeLa cells (Figure 7A). The functional cooperativity of the two agents is further confirmed in live-cell imaging analyses, where low doses of both drugs in combination were sufficient to strongly inhibit proliferation, in contrast to the application of the same doses as single treatments (Figure 7B). Quantification of these effects with the Chou–Talalay method [34] indicates that the combination of CPX with DCA is synergistic (combination index, CI < 1) over a broad concentration range (Figure 7C, upper left panel).

We extended these analyses to further combinations of CPX with either other glycolysis inhibitors (2-deoxy-D-glucose, 2-DG; 6-aminonicotinamide, 6-AN) or OXPHOS inhibitors (antimycin A; rotenone; metformin) in HeLa and SiHa cells. Consistent with CPX acting as an OXPHOS inhibitor, CPX synergizes not only with DCA but also with the glycolysis inhibitors 2-DG and 6-AN (CI < 1). In contrast, combinations of CPX with the OXPHOS inhibitors antimycin A, rotenone, and metformin act at most additively (CI around 1) (Figure 7C).

## 4. Discussion

Despite the availability of potent prophylactic vaccines protecting against infections with the majority of oncogenic HPV types, cervical cancer is expected to remain a major health burden in the upcoming years, especially in developing countries, and novel treatment options are urgently required [1,3]. CPX has been shown to possess promising anticancer potential in several preclinical cancer models [11]. We recently found that CPX blocks expression of the HPV oncogenes and can induce senescence or apoptosis in cervical cancer cells [8]. In the present work, we show that the decision of cervical cancer cells between these different phenotypic outcomes is critically dependent on glucose availability and is linked to the capacity of CPX to act both as an OXPHOS inhibitor and iron chelator. Moreover, we provide a rationale to combine CPX with glycolysis inhibitors to synergistically block the proliferation of cervical cancer cells.

Proteome analyses of HPV16-positive SiHa cervical cancer cells reveal that CPX downregulates factors involved in OXPHOS, in line with its iron-chelating potential [35], and stimulates factors involved in glycolysis. These findings are also supported by a recent study showing that CPX inhibits mitochondrial respiration and promotes the glycolysis rate in colorectal cancer cells [22]. Due to OXPHOS inhibition, CPX-treated cells may react particularly sensitive to glucose scarcity. Indeed, the induction of apoptosis in cervical cancer cells by CPX is strongly glucose-dependent, in that the pro-apoptotic activity of CPX is facilitated by limiting glucose availability and blocked by increasing glucose supply. Moreover, we show that the OXPHOS inhibitors antimycin A, rotenone, and metformin, which do not chelate iron, show the same glucose-dependent apoptosis induction in cervical cancer cells, supporting the notion that the pro-apoptotic function of CPX is due to its capacity to block OXPHOS.

Compared to cancer therapies using single agents, rational combinations of two or more drugs can be advantageous, as they may allow a reduction of the respective doses of the single drugs, leading to reduced side effects and a decreased risk of emerging drug resistance. In view of our data indicating that CPX-induced apoptosis in cervical cancer cells is linked to OXPHOS inhibition, we reasoned that concomitant targeting of glycolysis could cooperatively lead to even more pronounced anti-proliferative effects. Indeed, we found that combination treatments of CPX with different glycolysis inhibitors exhibit strong synergistic effects. This result is also interesting under the aspect that the systemic administration of iron chelators in the clinic is restricted due to dose-dependent severe side effects, such as gastrointestinal toxicity, which also has been observed for high CPX doses in a phase 1 study in patients with hematologic malignancies [49]. Similarly, the use of glycolysis inhibitors is restricted by dose-dependent toxicities, e.g., due to neuropathy [50,51]. The synergistic anti-proliferative effects, as observed in our study, indicate that a rational combination of CPX with a glycolysis inhibitor could allow a marked dose reduction of both drugs. Moreover, under therapeutic aspects it is noteworthy that CPX, in contrast to many other iron chelators, can be applied topically. This route of administration may circumvent side effects associated with the systemic application of iron chelators and could be particularly interesting in the context of HPV-linked (pre-) neoplastic lesions, which are typically located in the mucosa or skin [52,53] and are accessible for topical treatment.

Interestingly, in contrast to its pro-apoptotic activity, the pro-senescent activity of CPX is not shared by antimycin A, rotenone, or metformin, indicating that senescence induction by CPX is not due to OXPHOS inhibition. In line, CPX also efficiently induces senescence under increased glucose availability. Rather, we found that the pro-senescent activity of CPX is shared by other, structurally unrelated iron chelators, indicating that senescence induction by CPX is mediated by iron deprivation. This is further corroborated by our previous observation that CPX-induced senescence in cervical cancer cells is efficiently counteracted by supplementing iron [8].

Interestingly, HPV oncogene expression is not only strongly downregulated by CPX [8], but also by the OXPHOS inhibitor metformin [30] and by chronic hypoxia [28]. In all these cases, *E6/E7* repression is efficiently counteracted by increasing glucose supply, indicating that the HPV oncogene expression is very vulnerable to energy depletion. This also holds true for treating HPV-positive cancer cells with glycolysis inhibitors, such as 2-DG, which also effectively represses viral *E6/E7* expression [54]. It is well known that the HPV oncogenes can modulate the apoptosis and senescence response of cervical cancer cells [3]. However, it seems unlikely that the downregulation of *E6/E7* is essential for CPX-induced senescence or apoptosis, since HPV-negative cells can also show the same phenotypes under CPX treatment [8,11]. In addition, apoptosis and senescence induction in response to *E6/E7* inhibition are closely linked to the reconstitution of p53 through evasion from E6-mediated p53 degradation [55], however, both CPX-induced apoptosis and senescence appear to be independent of p53 and can be observed in loss-of-function p53 mutant cells [8,15].

Collectively, the findings of this study unravel the shift between senescence and apoptosis induction in CPX-treated cervical cancer cells. Besides providing biological insights into this regulation, these results could also be of therapeutic relevance for the current considerations to repurpose CPX for cancer treatment [11]. Similar to the clearance of apoptotic cancer cells [56], the induction of senescence in tumor cells, in principle, could be of therapeutic benefit by irreversibly blocking the growth of cancer cells [24]. However, senescence can be a double-edged sword under therapeutic aspects. Senescent cancer cells remain biologically active and secrete SASP factors which differ in their composition dependent on the cell type and the pro-senescent stimulus. Notably, these SASP factors can counteract or promote tumor progression in a non-cell-autonomous manner [57]. In the latter therapeutically non-favorable case, senescent cancer cells can lead to the suppression of antitumor immunity [58], provide non-senescent cancer cells with enhanced invasion [59] and metastasis [57] capacities, and lead to increased resistance towards chemotherapy [60,61]. It thus will be particularly interesting in future studies to investigate the possible influence of the CPX-induced SASP on the growth behavior and therapy resistance of proliferating cervical cancer cells.

## 5. Conclusions

In the present work, we provide novel insights into the phenotypic responses of cervical cancer cells towards CPX, which is currently under discussion to be repurposed as an anticancer drug. We show that CPX can block the growth of cervical cancer cells through two different anti-proliferative mechanisms, namely the induction of senescence and apoptosis. The switch between these phenotypic responses of cervical cancer cells is dependent on glucose availability. When glucose supply is limited, CPX induces apoptosis, an effect which is linked to its activity as an OXPHOS inhibitor. However, at increased glucose availability, CPX induces cellular senescence, an effect which is linked to its activity as an iron chelator. This bifunctionality of CPX to induce either senescence or apoptosis could influence the therapeutic outcome of CPX treatment. Moreover, we provide a rationale to therapeutically apply CPX together with glycolysis inhibitors, since this combination leads to synergistic pro-apoptotic effects in cervical cancer cells.

## Data Availability

The proteomics data are deposited to the ProteomeXchange Consortium via the PRIDE partner repository with the dataset identifier PXD011095.

## Supplementary Materials

The following are available online at https://www.mdpi.com/article/10.3390/cancers13194995/s1, Figure S1: Uncropped blots and quantification of immunoblots, Figure S2: Quantification of colony formation assays, Figure S3: Gene symbols and log2FC values, Figure S4: Colony forming capacity and senescence induction after 24 to 96 h CPX treatment, Figure S5: Regulation of senescence and cell cycle related proteins by CPX, Figure S6: Modulation of senescence-associated genes in CPX-treated cervical cancer cells.
