# Supplementary material for: Delineating the Switch between Senescence and Apoptosis in Cervical Cancer Cells under Ciclopirox Treatment
Source: Cancers (Basel). 2021 Oct 5;13(19):4995. doi: 10.3390/cancers13194995 (PMC8508512; doi:10.3390/cancers13194995)
Supplement: Supplementary file 1 [file cancers-13-04995-s001.zip › cancers-1409444-supplementary.pdf]

Supplementary Material

Delineating the Switch Between Senescence and Apoptosis in Cervical Cancer Cells Under Ciclopirox Treatment

Anja L. Herrmann, Bianca J. Kuhn, Angela Holzer, Jeroen Krijgsveld, Karin Hoppe-Seyler and Felix Hoppe-Seyler

Supplementary Figures S1-S6

Related to Figure 1B:

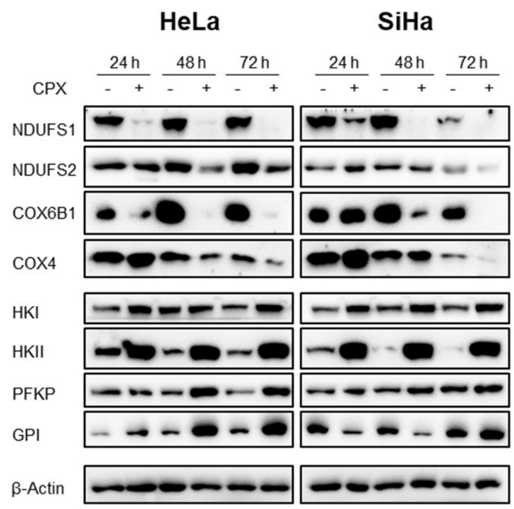

Uncropped original blots corresponding to Figure 1B:

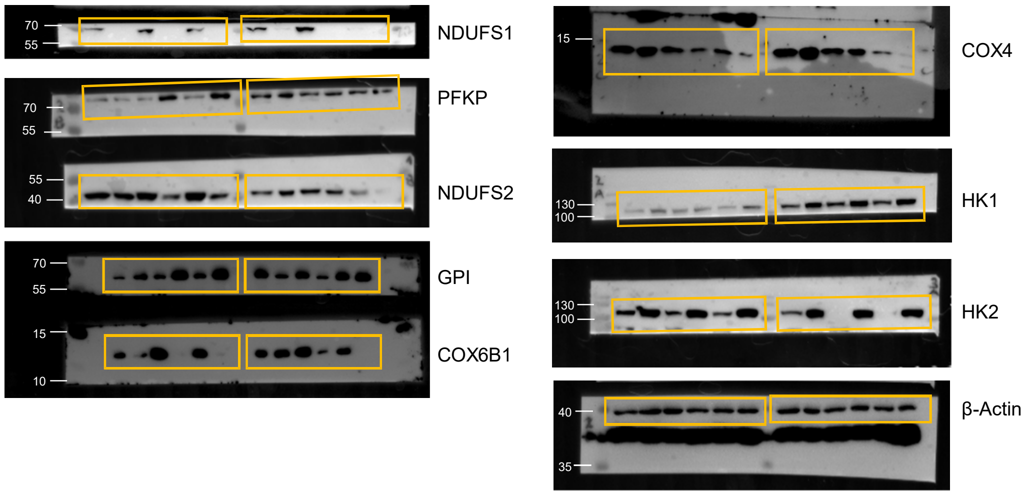

Densitometric analyses of blots in Figure 1B, upper values show raw densitometric readings, lower values are normalized to the sample written in **blue** (if possible the first lane):

	HeLa						SiHa						
Lane	1	2	3	4	5	6	1	2	3	4	5	6	
NDUFS1	**22548179**	1759790	24015108	751335	23104815	0	**26719300**	9697016	28769229	369607	7200752	0	raw values
	**1.00**	0.08	1.07	0.03	1.02	0.00	**1.00**	0.36	1.08	0.01	0.27	0.00	relative values
NDUFS2	**19742522**	19368765	24028421	12337744	27291714	15449572	**11103309**	13804915	13613380	10988894	7179338	2118891	raw values
	**1.00**	0.98	1.22	0.62	1.38	0.78	**1.00**	1.24	1.23	0.99	0.65	0.19	relative values
COX6B1	**17111158**	9216187	45951191	2325577	31258735	1238163	**21766643**	28888128	38009170	9262187	23102350	0	raw values
	**1.00**	0.54	2.69	0.14	1.83	0.07	**1.00**	1.33	1.75	0.43	1.06	0.00	relative values
COX4	**26460472**	29208563	19064915	8458631	11738066	4111246	**28587078**	35688706	21984693	17551208	5797196	564263	raw values
	**1.00**	1.10	0.72	0.32	0.44	0.16	**1.00**	1.25	0.77	0.61	0.20	0.02	relative values
HKI	**13061966**	23201815	19041501	19595522	14277158	24449250	**11514844**	19680522	15335865	22753572	12994258	21689622	raw values
	**1.00**	1.78	1.46	1.50	1.09	1.87	**1.00**	1.71	1.33	1.98	1.13	1.88	relative values
HKII	**17498137**	34498128	10505480	32163401	9603581	35677007	**9785874**	33774300	3169175	35092401	2301134	32948158	raw values
	**1.00**	1.97	0.60	1.84	0.55	2.04	**1.00**	3.45	0.32	3.59	0.24	3.37	relative values
PFKP	**14504258**	12464602	11114480	23950744	9102702	21949208	**12583723**	13284258	13395844	18204622	17520966	20141622	raw values
	**1.00**	0.86	0.77	1.65	0.63	1.51	**1.00**	1.06	1.06	1.45	1.39	1.60	relative values
GPI	**3345347**	11872602	8001752	28221078	10872480	29570836	**19226572**	8436752	16207744	5825903	18783057	24294957	raw values
	**1.00**	3.55	2.39	8.44	3.25	8.84	**1.00**	0.44	0.84	0.30	0.98	1.26	relative values
Actin	**15686744**	20234593	20413371	16619643	17592007	17713007	**19541522**	17069229	16622744	17329208	15877622	15651794	raw values
	**1.00**	1.29	1.30	1.06	1.12	1.13	**1.00**	0.87	0.85	0.89	0.81	0.80	relative values

Figure S1. Uncropped blots and quantification of immunoblots. Uncropped original immunoblots including molecular weight markers are depicted. Immunoblots were quantified densitometrically (raw values) and were normalized to the sample written in blue (relative values). Continued on next pages.

Related to Figure 2B:

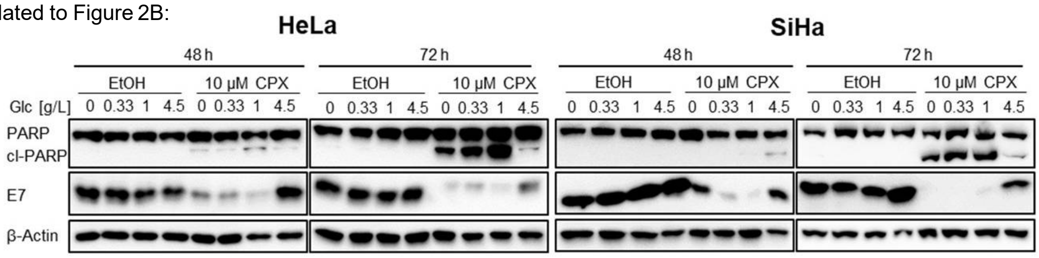

Uncropped original blots corresponding to Figure 2B:

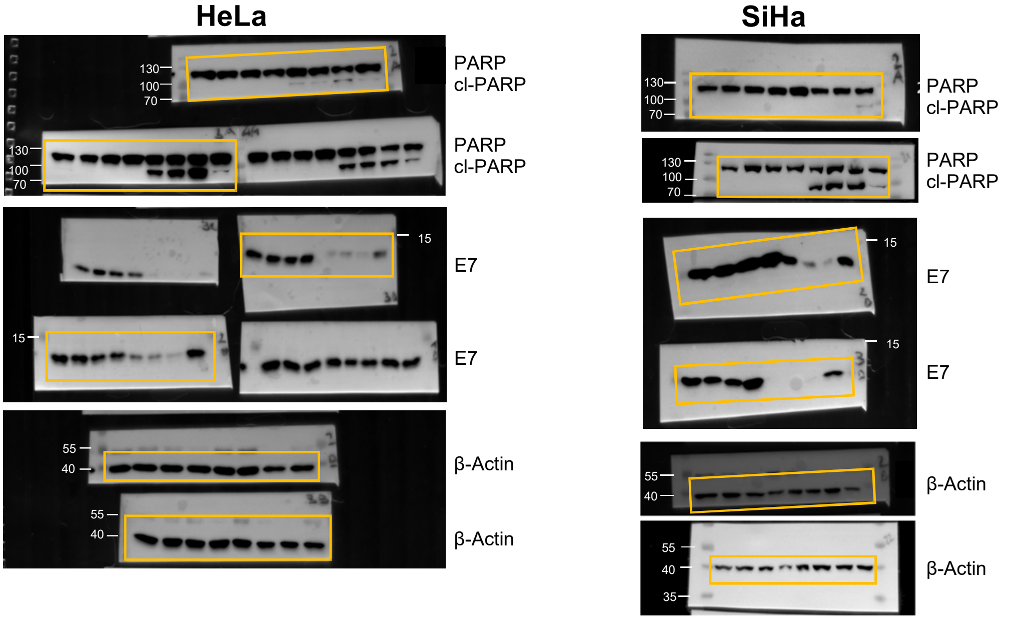

Densitometric analyses of blots in Figure 2B, upper values show raw densitometric readings, lower values are normalized to the sample written in **blue** (if possible the first lane):

	HeLa (48 h)								
Lane	1	2	3	4	5	6	7	8	
**cleaved PARP**	0	0	0	0	**3009690**	1770205	10361510	1565426	raw values
	0.00	0.00	0.00	0.00	**1.00**	0.59	3.44	0.52	relative values
**E7**	**33649848**	32915969	25328484	23331362	11122279	8336773	6515175	33672484	raw values
	**1.00**	0.98	0.75	0.69	0.33	0.25	0.19	1.00	relative values
**Actin**	**20745380**	22613087	21758622	22423208	23291915	24183401	16113723	19080673	raw values
	**1.00**	1.09	1.05	1.08	1.12	1.17	0.78	0.92	relative values

	HeLa (72 h)								
Lane	1	2	3	4	5	6	7	8	
**cleaved PARP**	0	0	0	0	**17712451**	27823128	37788350	1853669	raw values
	0.00	0.00	0.00	0.00	**1.00**	1.57	2.13	0.10	relative values
**E7**	**33001990**	31867605	29028291	31010413	2117820	4546246	3019719	13866300	raw values
	**1.00**	0.97	0.88	0.94	0.06	0.14	0.09	0.42	relative values
**Actin**	**27834271**	28000128	26720300	28391664	25737421	19916815	21044765	23113492	raw values
	**1.00**	1.01	0.96	1.02	0.92	0.72	0.76	0.83	relative values

	SiHa (48 h)								
Lane	1	2	3	4	5	6	7	8	
**cleaved PARP**	0	0	0	0	0	0	0	**9731187**	raw values
	0	0	0	0	0	0	0	**1**	relative values
**E7**	**34659371**	39140271	44445534	47378040	17434664	2639719	1612749	17325179	raw values
	**1.00**	1.13	1.28	1.37	0.50	0.08	0.05	0.50	relative values
**Actin**	**23090401**	22743836	19428714	15661815	18324229	18913350	21584421	13690208	raw values
	**1.00**	0.98	0.84	0.68	0.79	0.82	0.93	0.59	relative values

	SiHa (72 h)								
Lane	1	2	3	4	5	6	7	8	
**cleaved PARP**	0	0	0	0	**16303986**	27134472	26534957	4054125	raw values
	0.00	0.00	0.00	0.00	**1.00**	1.66	1.63	0.25	relative values
**E7**	**32582149**	26220492	26926200	39257120	0	0	681021	17310693	raw values
	**1.00**	0.80	0.83	1.20	0.00	0.00	0.02	0.53	relative values
**Actin**	**17613794**	19535087	16659844	10572480	19972622	24443744	21700501	20419572	raw values
	**1.00**	1.11	0.95	0.60	1.13	1.39	1.23	1.16	relative values

Figure S1 continued.

Related to Figure 3C:

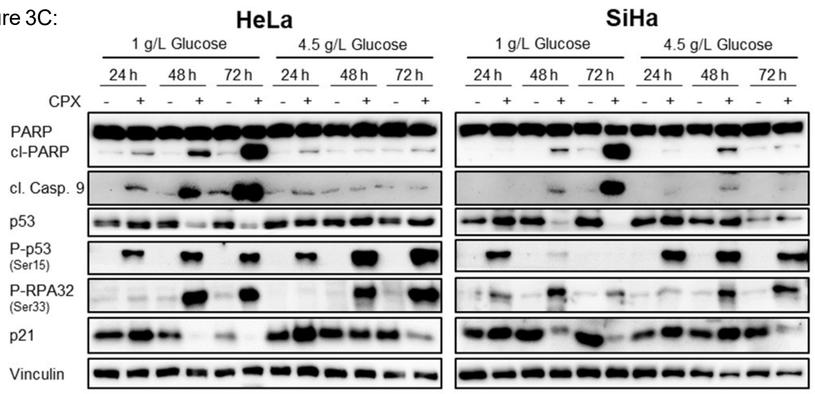

Uncropped original blots corresponding to Figure 3C:

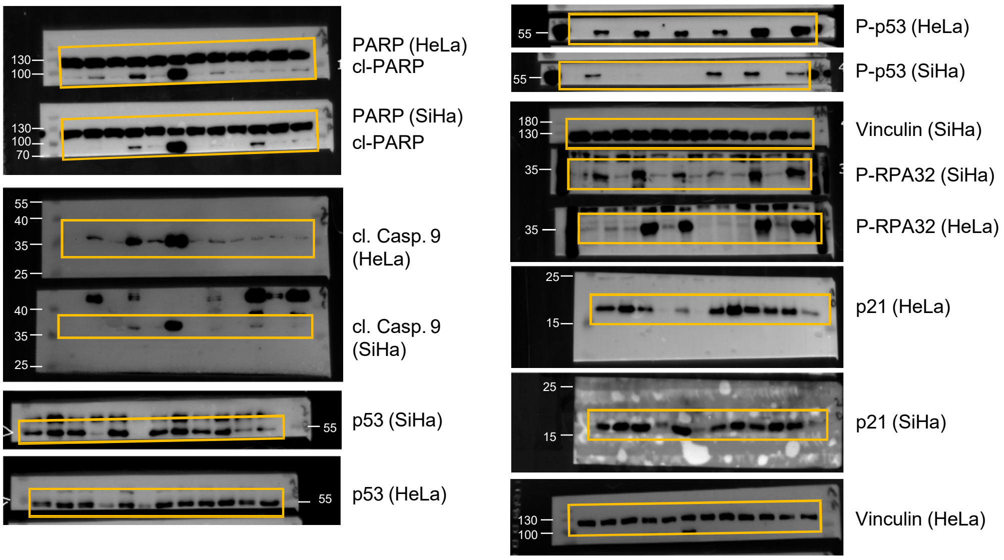

Densitometric analyses of blots in Figure 3C, upper values show raw densitometric readings, lower values are normalized to the sample written in **blue** (if possible the first lane):

	HeLa												
Lane	1	2	3	4	5	6	7	8	9	10	11	12	
**cleaved PARP**	1971134	**7107581**	1822062	17755815	7636874	43241057	4556104	5074731	1298062	1690548	1569548	3715832	raw values
	0.28	**1.00**	0.26	2.50	1.07	6.08	0.64	0.71	0.18	0.24	0.22	0.52	relative values
**cleaved Caspase 9**	0	**7624874**	3668640	23902563	12342037	45790877	5956974	5238731	2956619	2998740	2140497	1959548	raw values
	0.00	**1.00**	0.48	3.13	1.62	6.01	0.78	0.69	0.39	0.39	0.28	0.26	relative values
**p53**	**17993350**	22455765	20504472	6995267	19034693	7980731	22918401	21034643	22523472	25339421	17711643	22345815	raw values
	**1.00**	1.25	1.14	0.39	1.06	0.44	1.27	1.17	1.25	1.41	0.98	1.24	relative values
**P-p53**	0	**16344430**	0	22909229	0	21274037	0	18661279	0	39537936	0	41514563	raw values
	0.00	**1.00**	0.00	1.40	0.00	1.30	0.00	1.14	0.00	2.42	0.00	2.54	relative values
**P-RPA32**	**1621062**	2103548	1957305	33931635	3531690	24540543		491607	424021	29116806	4105347	39468706	raw values
	**1.00**	1.30	1.21	20.93	2.18	15.14	0.00	0.30	0.26	17.96	2.53	24.35	relative values
**p21**	**25780513**	33933676	19784392	1930991	8559723	2149719	26589342	44979697	34185413	25959220	26217685	9781966	raw values
	**1.00**	1.32	0.77	0.07	0.33	0.08	1.03	1.74	1.33	1.01	1.02	0.38	relative values
**Vinculin**	**24040087**	20055673	22129794	17124016	16854208	19148208	20521501	24382329	22174380	20166894	13719773	15886430	raw values
	**1.00**	0.83	0.92	0.71	0.70	0.80	0.85	1.01	0.92	0.84	0.57	0.66	relative values

	SiHa												
Lane	1	2	3	4	5	6	7	8	9	10	11	12	
**cleaved PARP**	0	0	0	**8574359**	1011062	43397735	0	214849	0	10535844	327678	771527	raw values
	0.00	0.00	0.00	**1.00**	0.12	5.06	0.00	0.03	0.00	1.23	0.04	0.09	relative values
**cleaved Caspase 9**	0	0	0	**5325731**	0	30406886	0	1040920	0	2756054	0	86607	raw values
	0.00	0.00	0.00	**1.00**	0.00	5.71	0.00	0.20	0.00	0.52	0.00	0.02	relative values
**p53**	**11883551**	24070300	22106765	1913104	25740451	2141719	20099229	23775158	17515279	20327451	6475702	6295338	raw values
	**1.00**	2.03	1.86	0.16	2.17	0.18	1.69	2.00	1.47	1.71	0.54	0.53	relative values
**P-p53**	0	**21357765**	0	2644790	0	0	0	28104078	0	29542593	0	26279714	raw values
	0.00	**1.00**	0.00	0.12	0.00	0.00	0.00	1.32	0.00	1.38	0.00	1.23	relative values
**P-RPA32**	3003619	**11127380**	1095234	19670886	1927134	7733652	1361891	4059539	1310891	18816007	1065991	22360999	raw values
	0.27	**1.00**	0.10	1.77	0.17	0.70	0.12	0.36	0.12	1.69	0.10	2.01	relative values
**p21**	**19420815**	28877907	31037978	9528037	29607877	6249116	16912836	25429492	18889078	26037200	22335442	7893288	raw values
	**1.00**	1.49	1.60	0.49	1.52	0.32	0.87	1.31	0.97	1.34	1.15	0.41	relative values
**Vinculin**	**28483250**	22097057	25511300	24269593	27161421	26555714	25794179	21588936	18845815	12184087	17353329	12830137	raw values
	**1.00**	0.78	0.90	0.85	0.95	0.93	0.91	0.76	0.66	0.43	0.61	0.45	relative values

Figure S1 continued.

Related to Figure 4A:

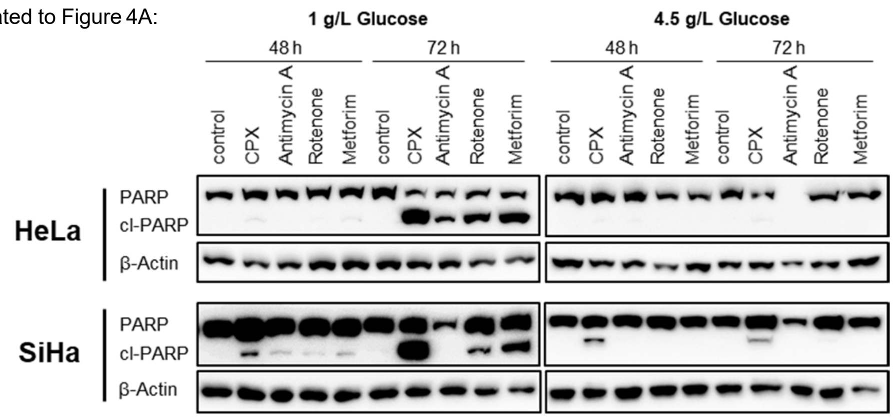

Uncropped original blots corresponding to Figure 4A:

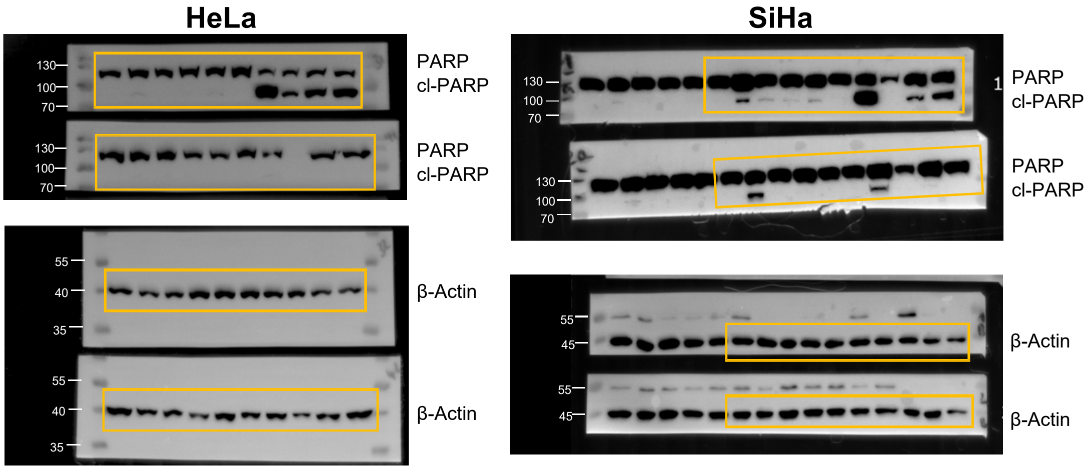

Densitometric analyses of blots in Figure 4A, upper values show raw densitometric readings, lower values are normalized to the sample written in blue (if possible the first lane):

	HeLa, 1 g/L Glucose										
Lane	1	2	3	4	5	6	7	8	9	10	
cleaved PARP	0	429435	0	0	386607	0	**35486229**	13169915	21073037	27033350	raw values
	0.00	0.01	0.00	0.00	0.01	0.00	**1.00**	0.37	0.59	0.76	relative values
Actin	**19348522**	13645915	15949865	23794836	24373664	22803250	20111886	17926936	13817208	13595794	raw values
	**1.00**	0.71	0.82	1.23	1.26	1.18	1.04	0.93	0.71	0.70	relative values

	HeLa, 4.5 g/L Glucose										
Lane	1	2	3	4	5	6	7	8	9	10	
cleaved PARP	0	0	0	0	0	0	0	0	0	0	raw values
	0.00	0.00	0.00	0.00	0.00	0.00	0.00	0.00	0.00	0.00	relative values
Actin	**23260693**	14342844	13728087	9601945	17967551	13880016	15566016	8268874	15808501	22409350	raw values
	**1.00**	0.62	0.59	0.41	0.77	0.60	0.67	0.36	0.68	0.96	relative values

	SiHa, 1 g/L Glucose										
Lane	1	2	3	4	5	6	7	8	9	10	
cleaved PARP	0	6695338	1502548	740577	1292962	0	**43241836**	0	10492551	22191451	raw values
	0.00	0.15	0.03	0.02	0.03	0.00	**1.00**	0.00	0.24	0.51	relative values
Actin	**23452522**	25144179	20887522	21768865	20588572	21733986	18969279	18515158	14337087	13259087	raw values
	**1.00**	1.07	0.89	0.93	0.88	0.93	0.81	0.79	0.61	0.57	relative values

	SiHa, 4.5 g/L Glucose										
Lane	1	2	3	4	5	6	7	8	9	10	
cleaved PARP	0	14097158	0	0	0	0	**9911673**	0	564092	0	raw values
	0.00	1.42	0.00	0.00	0.00	0.00	**1.00**	0.00	0.06	0.00	relative values
Actin	**20255087**	17816258	23556208	21391966	19896794	17116309	17501744	18125815	20726522	10411480	raw values
	**1.00**	0.88	1.16	1.06	0.98	0.85	0.86	0.89	1.02	0.51	relative values

Figure S1 continued.

Related to Figure 6A:

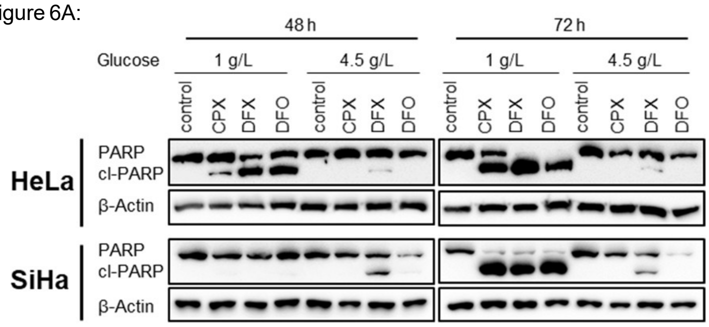

Uncropped original blots corresponding to Figure 6A:

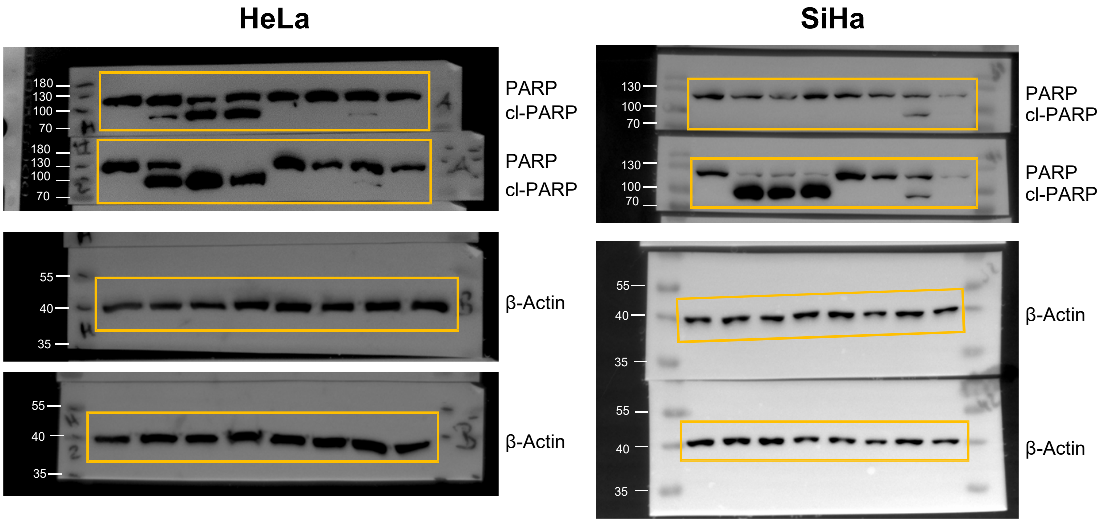

Densitometric analyses of blots in Figure 6A, upper values show raw densitometric readings, lower values are normalized to the sample written in blue (if possible the first lane):

	HeLa (48 h)								
Lane	1	2	3	4	5	6	7	8	
cleaved PARP	0	**5642731**	19181572	23985329	0	0	988598	0	raw values
	0.00	**1.00**	3.40	4.25	0.00	0.00	0.18	0.00	relative values
Actin	**17567179**	16956936	19755836	25217250	28011785	22854714	27773200	27218321	raw values
	**1.00**	0.97	1.12	1.44	1.59	1.30	1.58	1.55	relative values

	HeLa (72 h)								
Lane	1	2	3	4	5	6	7	8	
cleaved PARP	0	**35323229**	47883614	30030593	0	0	1754477	0	raw values
	0.00	**1.00**	1.36	0.85	0.00	0.00	0.05	0.00	relative values
Actin	**17485865**	24709279	20943329	26828421	26312643	27985472	30211028	22624028	raw values
	**1.00**	1.41	1.20	1.53	1.50	1.60	1.73	1.29	relative values

	SiHa (48 h)								
Lane	1	2	3	4	5	6	7	8	
cleaved PARP	0	0	0	0	0	0	**13124652**	166849	raw values
	0.00	0.00	0.00	0.00	0.00	0.00	**1.00**	0.01	relative values
Actin	**20744158**	22011522	18887572	21872522	23021643	15652208	21528865	16795744	raw values
	**1.00**	1.06	0.91	1.05	1.11	0.75	1.04	0.81	relative values

	SiHa (72 h)								
Lane	1	2	3	4	5	6	7	8	
cleaved PARP	0	**36810321**	30545907	35981886	0	0	4832246	0	raw values
	0.00	**1.00**	0.83	0.98	0.00	0.00	0.13	0.00	relative values
Actin	**19370894**	20878187	19990480	15132652	16388602	12021338	17644359	14072581	raw values
	**1.00**	1.08	1.03	0.78	0.85	0.62	0.91	0.73	relative values

Figure S1 continued.

Related to Figure 7A:

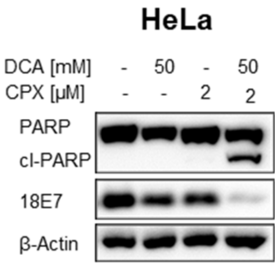

Uncropped original blots corresponding to Figure 7A:

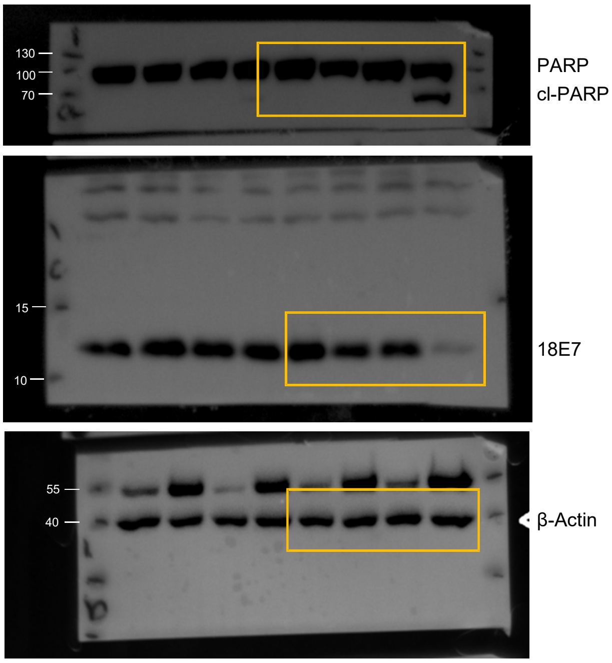

Densitometric analyses of blots in Figure 7A, upper values show raw densitometric readings, lower values are normalized to the sample written in blue (if possible the first lane):

Lane		1	2	3	4	
**cleaved PARP**		0	0	0	**11962673**	raw values
		0.00	0.00	0.00	**1.00**	relative values
**E7**		**28505543**	20111765	21097886	3561004	raw values
		**1.00**	0.71	0.74	0.12	relative values
**Actin**		**20171179**	20939421	21368714	23990543	raw values
		**1.00**	1.04	1.06	1.19	relative values

Figure S1 continued.

Related to Figure S5:

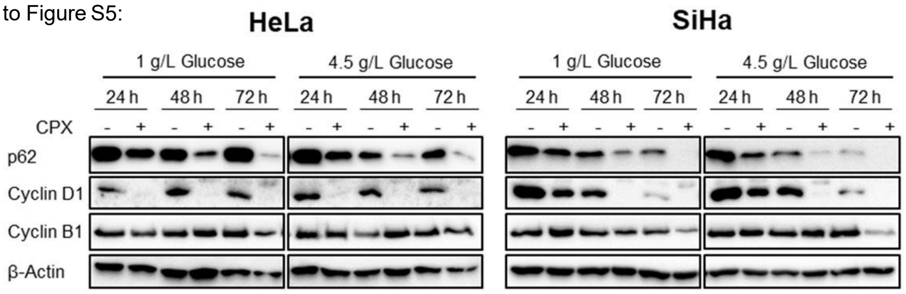

Uncropped original blots corresponding to Figure S5:

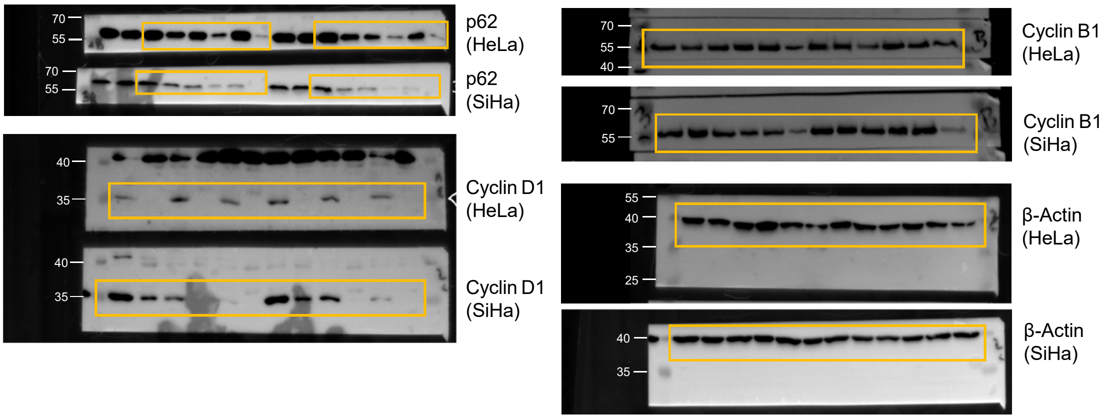

Densitometric analyses of blots in Figure S5, upper values show raw densitometric readings, lower values are normalized to the sample written in **blue** (if possible the first lane)

	HeLa, 1 g/L Glucose						HeLa, 4.5 g/L Glucose						
Lane	1	2	3	4	5	6	7	8	9	10	11	12	
p62	**31872250**	21516279	24138986	11332309	31243593	4699974	**34791250**	21330936	14761794	5382681	18563350	2294790	raw values
	**1.00**	0.68	0.76	0.36	0.98	0.15	**1.00**	0.61	0.42	0.15	0.53	0.07	relative values
Cyclin D1	**11907915**	0	18566693	0	15405522	0	**20953179**	868920	15768037	0	12751309	0	raw values
	**1.00**	0.00	1.56	0.00	1.29	0.00	**1.00**	0.04	0.75	0.00	0.61	0.00	relative values
Cyclin B1	**18088158**	12861137	17031329	18942329	20323986	10771359	**19641522**	17020865	11336723	20431815	16728865	13487551	raw values
	**1.00**	0.71	0.94	1.05	1.12	0.60	**1.00**	0.87	0.58	1.04	0.85	0.69	relative values
Actin	**22775057**	20699643	24172543	30957907	20940179	14792037	**24477836**	21988765	18892815	21640108	17585744	12437137	raw values
	**1.00**	0.91	1.06	1.36	0.92	0.65	**1.00**	0.90	0.77	0.88	0.72	0.51	relative values

	SiHa, 1 g/L Glucose						SiHa, 4.5 g/L Glucose						
Lane	1	2	3	4	5	6	7	8	9	10	11	12	
p62	**25680664**	16802572	15461622	5073610	8480874	298192	**25376321**	12985087	9394773	1216062	2396569	0	raw values
	**1.00**	0.65	0.60	0.20	0.33	0.01	**1.00**	0.51	0.37	0.05	0.09	0.00	relative values
Cyclin D1	**32480785**	15690380	14479430	212364	2466205	0	**30445128**	16631673	18243087	878506	4884731	0	raw values
	**1.00**	0.48	0.45	0.01	0.08	0.00	**1.00**	0.55	0.60	0.03	0.16	0.00	relative values
Cyclin B1	**15906258**	17872622	14636258	11525309	11534187	6037146	**19334572**	18618279	17992158	18628572	21797936	5626024	raw values
	**1.00**	1.12	0.92	0.72	0.73	0.38	**1.00**	0.96	0.93	0.96	1.13	0.29	relative values
Actin	**23812593**	22731543	23151593	23242421	24538028	21050543	**20715421**	16818936	15873279	17762401	18735522	20037986	raw values
	**1.00**	0.95	0.97	0.98	1.03	0.88	**1.00**	0.81	0.77	0.86	0.90	0.97	relative values

Figure S1 continued.

Quantification of CFAs in Figure 3:

HeLa	72 h EtOH				72 h CPX				96 h EtOH				96 h CPX			
Glucose	0 g/L	0.33 g/L	1 g/L	4.5 g/L	0 g/L	0.33 g/L	1 g/L	4.5 g/L	0 g/L	0.33 g/L	1 g/L	4.5 g/L	0 g/L	0.33 g/L	1 g/L	4.5 g/L
% area	80.00	85.07	86.16	88.00	32.42	18.98	14.19	24.85	84.51	89.18	90.13	88.48	4.79	2.25	0.41	8.69
colony count	n.d.	n.d.	n.d.	n.d.	726	417	323	547	n.d.	n.d.	n.d.	n.d.	159	97	23	293

SiHa	72 h EtOH				72 h CPX				96 h EtOH				96 h CPX			
Glucose	0 g/L	0.33 g/L	1 g/L	4.5 g/L	0 g/L	0.33 g/L	1 g/L	4.5 g/L	0 g/L	0.33 g/L	1 g/L	4.5 g/L	0 g/L	0.33 g/L	1 g/L	4.5 g/L
% area	85.23	96.00	99.19	99.96	25.20	21.30	1.46	0.46	84.25	93.13	99.13	99.37	0.55	0.02	0.00	0.03
colony count	n.d.	n.d.	n.d.	n.d.	518	485	32	15	n.d.	n.d.	n.d.	n.d.	32	1	0	1

Quantification of CFAs in Figure 5:

HeLa	1 g/L Glucose					4.5 g/L Glucose				
	control	CPX	AA	Rot	Met	control	CPX	AA	Rot	Met
% area	91.49	11.21	28.37	9.65	70.28	93.84	27.07	94.08	66.30	91.13
colony count	n.d.	401	932	398	n.d.	n.d.	713	n.d.	n.d.	n.d.

SiHa	1 g/L Glucose					4.5 g/L Glucose				
	control	CPX	AA	Rot	Met	control	CPX	AA	Rot	Met
% area	86.97	0.68	35.34	47.82	46.38	90.21	0.76	96.96	87.72	93.47
colony count	n.d.	53	995	n.d.	n.d.	n.d.	30	n.d.	n.d.	n.d.

Quantification of CFAs in Figure 6:

HeLa	1 g/L Glucose				4.5 g/L Glucose			
	control	CPX	DFX	DFO	control	CPX	DFX	DFO
% area	91.58	7.38	2.74	1.08	99.84	9.23	26.90	5.65
colony count	n.d.	254	87	66	n.d.	295	857	227

SiHa	1 g/L Glucose				4.5 g/L Glucose			
	control	CPX	DFX	DFO	control	CPX	DFX	DFO
% area	99.79	5.36	16.39	9.33	97.88	0.97	2.53	1.18
colony count	n.d.	218	412	345	n.d.	26	113	66

Quantification of CFAs in Figure S4:

HeLa	24 h CPX				48 h CPX				72 h CPX				96 h CPX			
Glucose	0 g/L	0.33 g/L	1 g/L	4.5 g/L	0 g/L	0.33 g/L	1 g/L	4.5 g/L	0 g/L	0.33 g/L	1 g/L	4.5 g/L	0 g/L	0.33 g/L	1 g/L	4.5 g/L
% area	76.06	75.16	73.87	75.15	56.73	57.05	59.94	62.35	32.42	18.98	14.19	24.85	4.79	2.25	0.41	8.69

SiHa	24 h CPX				48 h CPX				72 h CPX				96 h CPX			
Glucose	0 g/L	0.33 g/L	1 g/L	4.5 g/L	0 g/L	0.33 g/L	1 g/L	4.5 g/L	0 g/L	0.33 g/L	1 g/L	4.5 g/L	0 g/L	0.33 g/L	1 g/L	4.5 g/L
% area	40.36	68.83	75.29	67.24	12.02	31.39	4.82	8.17	25.20	21.30	1.46	0.46	0.55	0.02	0.00	0.03

Figure S2. Quantification of colony formation assays. The percentage of area covered with colonies was quantified for the CFAs depicted in Figures 3, 5, 6, and S2. Where possible also the number of colonies was quantified.

A

GO glycolytic process

Symbol	log2FC	Symbol	log2FC	Symbol	log2FC	Symbol	log2FC
HK2	1.52	NUP155	0.51	PGK2	0.18	ALDOA	-0.39
PFKFB3	1.16	NUP35	0.49	NUP50	0.17	TPI1	-0.40
HIF1A	1.05	NUP160	0.45	ZBTB7A	0.17	LDHA	-0.50
ENO2	0.76	RANBP2	0.41	NUPL2	0.12	EIF6	-0.51
ADPGK	0.75	OGDH	0.41	NUP188	0.07	GAPDH	-0.52
HK1	0.75	SEH1L	0.40	POM121C	0.01	PFKFB2	-0.58
NUP37	0.70	NUP43	0.40	SEC13	0.00	GALK1	-0.60
ACTN3	0.67	NUP98	0.36	NUP93	-0.06	PGM1	-0.65
NUP210	0.64	NUP214	0.32	NCOR1	-0.06	PFKM	-0.65
TPR	0.58	NDC1	0.30	PFKP	-0.06	PGAM1	-0.68
NUP153	0.57	NUP88	0.29	ALDOC	-0.06	PKM	-0.71
ARNT	0.57	AAAS	0.26	PFKL	-0.08	OGT	-0.77
NUP54	0.57	NUP205	0.25	PGK1	-0.13	ENO1	-0.79
NUP62	0.57	INSR	0.23	DHTKD1	-0.28	ECD	-0.82
NUP133	0.54	RAE1	0.20	TIGAR	-0.30	GPI	-0.83
NUP107	0.54	NUP85	0.20	PRKAG1	-0.31	STAT3	-1.47
NUP58	0.53	ENTPD5	0.18	PRKAA1	-0.36		

B

GO oxidative phosphorylation

Symbol	log2FC	Symbol	log2FC	Symbol	log2FC	Symbol	log2FC
ATP5MC3	1.27	PGK2	0.18	SHMT2	-0.13	NDUFA12	-0.63
ACTN3	0.67	COX4I1	0.15	UQCRC1	-0.17	NDUFA8	-0.67
SDHAF2	0.65	ATP5F1D	0.14	MSH2	-0.20	NDUFA7	-0.73
COQ9	0.63	UQCC2	0.14	NDUFB10	-0.20	COX7C	-0.86
PPIF	0.52	COX5A	0.11	NDUFB9	-0.22	CDK1	-0.88
DNAJC30	0.49	PDE12	0.09	COX5B	-0.26	NDUFA5	-0.89
SURF1	0.48	NIPSNAP2	0.09	NDUFC2	-0.33	PARK7	-0.96
COX7A2L	0.39	NDUFB6	0.06	UQCRB	-0.33	NDUFS3	-1.01
MT-ND4	0.39	CYCS	0.05	NDUFB8	-0.35	NDUFA13	-1.03
ABCD1	0.37	NDUFAF1	0.05	COX6C	-0.36	COX7A2	-1.04
ATP5F1A	0.33	RHOA	0.01	UQCRC2	-0.38	COX6B1	-1.16
UQCR10	0.32	NDUFB11	-0.01	UQCRFS1	-0.44	NDUFV1	-1.23
ATP5F1C	0.32	MT-ATP8	-0.01	NDUFA9	-0.47	NDUFS8	-1.27
ATP5F1E	0.31	NDUFB5	-0.04	CCNB1	-0.48	NDUFA4	-1.28
SDHA	0.31	MT-ND3	-0.04	NDUFB7	-0.50	NDUFA6	-1.30
ATP5F1B	0.27	VCP	-0.05	BID	-0.50	NDUFS1	-1.34
DLD	0.27	NDUFAB1	-0.06	NDUFB4	-0.51	NDUFS5	-1.37
MECP2	0.25	CYC1	-0.06	NDUFS2	-0.53	NDUFS7	-1.41
STOML2	0.25	NDUFS6	-0.10	NDUFB3	-0.55	NDUFV2	-1.49
COX15	0.24	UQCRH	-0.10	NDUFA11	-0.55	NDUFA2	-1.49
FXN	0.24	UQCRQ	-0.12	NDUFA10	-0.57	NDUFS4	-1.53
COA6	0.23	PGK1	-0.13	MT-CO2	-0.59		

Figure S3. Gene symbols and log2FC values. Gene symbols and average log2FC values of proteins encoded by genes contained in the gene sets "GO_glycolytic_process" (A) and "GO_oxidative_phosphorylation" (B), corresponding to Figure 1A. Color scale indicates log2FC: blue, log2FC = -1.5; yellow, log2FC = 0; red, log2FC = 1.5.

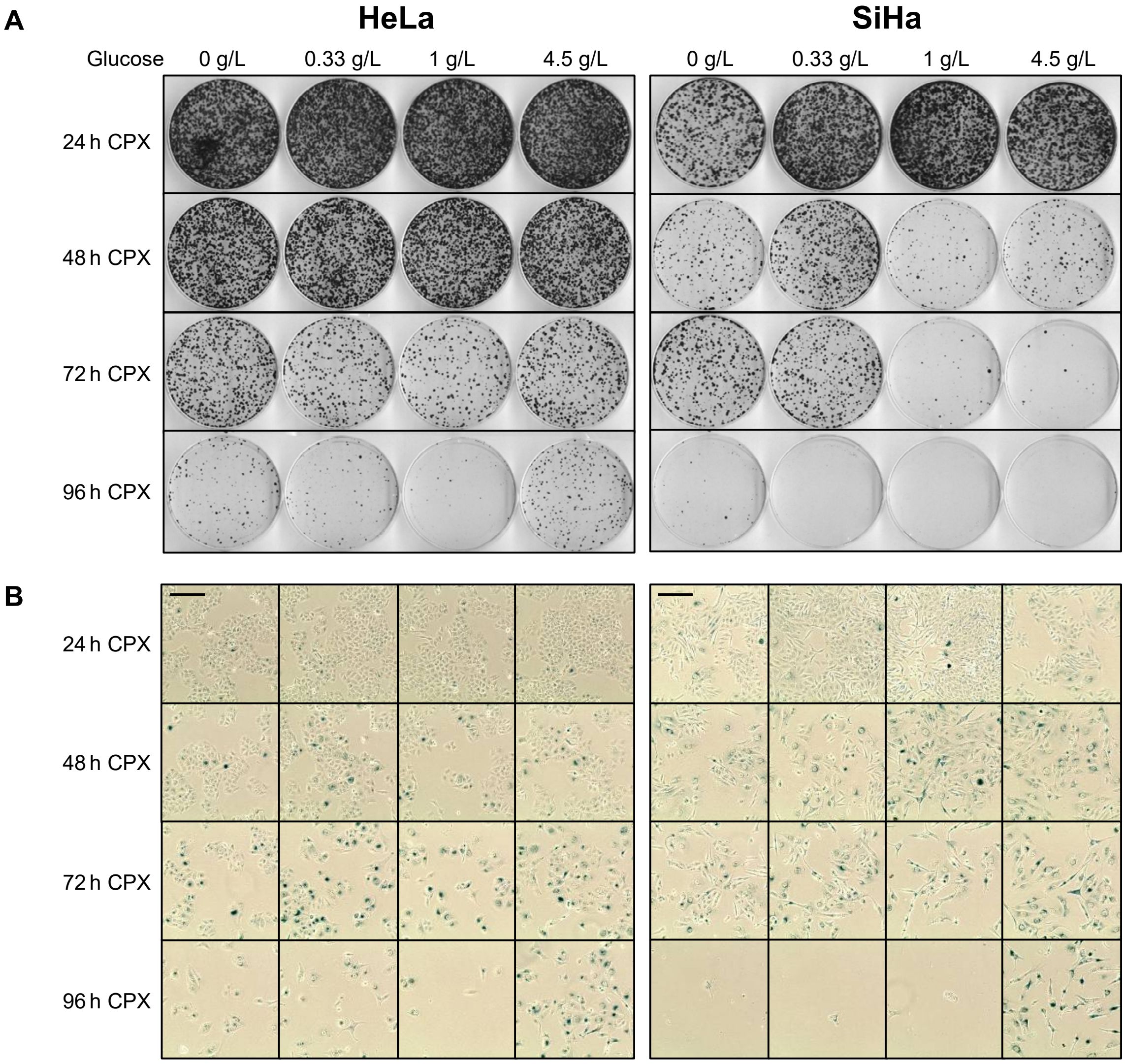

Figure S4. Colony forming capacity and senescence induction after 24 to 96 h CPX treatment. Colony formation assays (CFAs) (A) and senescence assays (B) of HeLa and SiHa cells treated with 10 µM CPX for 24 to 96 h under the indicated glucose concentrations. The 72 h and 96 h time points are also depicted in Figure 3. For CFAs, cells were grown in CPX-free medium under 1 g/L glucose for 12 days, fixed and stained. SA-β-gal assays were performed after release for 4 days. Scale bars: 200 µm.

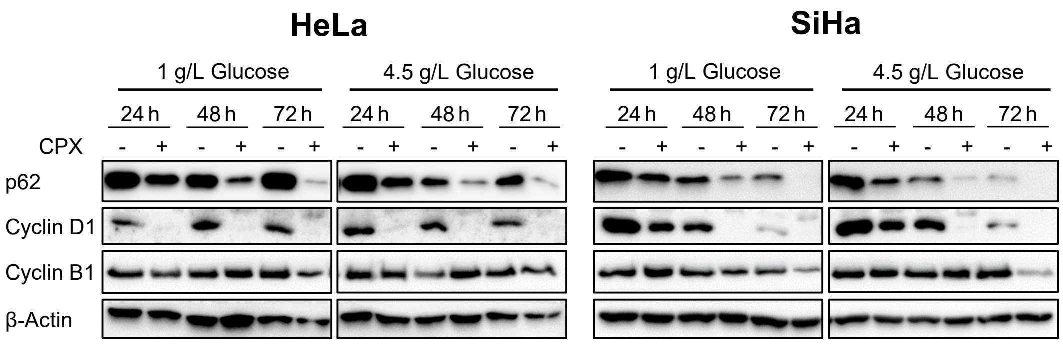

Figure S5. Regulation of senescence- and cell cycle-related proteins by CPX. Immunoblot analyses of HeLa and SiHa cells treated for 24, 48, or 72 h with 10 μM CPX (+) or solvent control (-), analyzing protein levels of p62, Cyclin D1 and Cyclin B1 upon cultivation under 1 g/L or 4.5 g/L glucose. β-Actin: representative loading control.

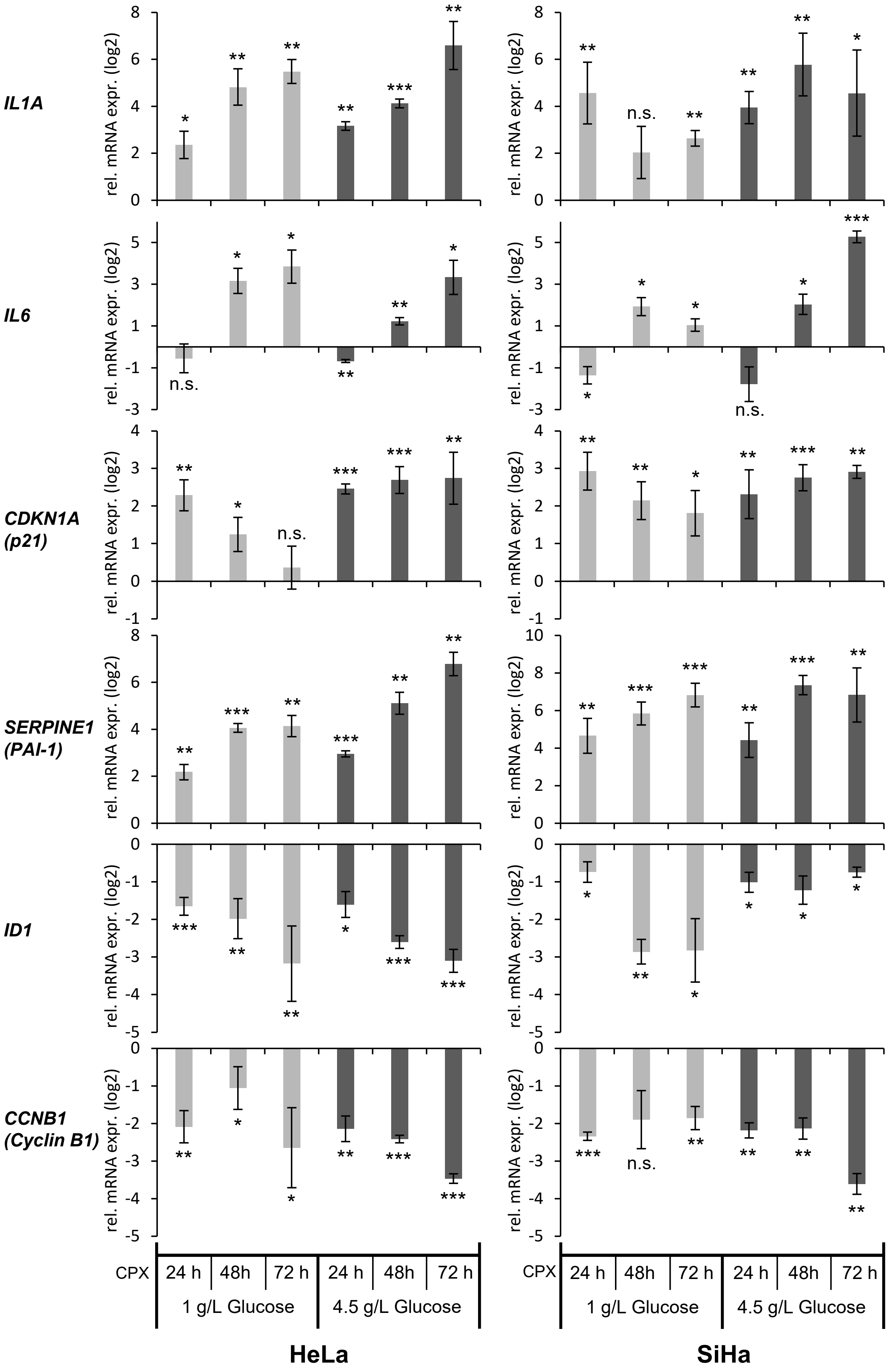

Figure S6. Modulation of senescence-associated genes in CPX-treated cervical cancer cells. qRT-PCR analyses determining relative mRNA expression levels of senescence-associated genes in HeLa (left panels) or SiHa (right panels) cells after 24, 48, or 72 h treatment with 10 μM CPX under 1 g/L or 4.5 g/L glucose. Expression levels were normalized to solvent control (EtOH) treated cells for each time point and are depicted as log2 fold changes. Error bars indicate standard deviations of 3-4 experiments. n.s., non-significant; * = $p \leq 0.05$; ** = $p \leq 0.01$. *** = $p \leq 0.001$.
